# Supragingival microbiome alternations as a consequence of smoking different tobacco types and its relation to dental caries

**DOI:** 10.1038/s41598-022-06907-z

**Published:** 2022-02-21

**Authors:** Farah Al-Marzooq, Sausan Al Kawas, Betul Rahman, Jenni A. Shearston, Hiba Saad, Dalenda Benzina, Michael Weitzman

**Affiliations:** 1 Department of Medical Microbiology and Immunology, College of Medicine and Health Sciences, UAE University, Al Ain, United Arab Emirates; 2 Department of Oral and Craniofacial Health Sciences, College of Dental Medicine, University of Sharjah, P.O.Box: 27272, Sharjah, United Arab Emirates; 3 Sharjah Institute for Medical Research, University of Sharjah, Sharjah, United Arab Emirates; 4 Department of Preventive and Restorative Dentistry, College of Dental Medicine, University of Sharjah, Sharjah, United Arab Emirates; 5 Department of Environmental Health Sciences, Mailman School of Public Health, Columbia University, New York, USA; 6 Department of Pediatrics, School of Medicine, New York University, New York, USA; 7 Department of Environmental Medicine, School of Medicine, New York University, New York, USA; 8 College of Global Public Health, New York University, New York, USA

**Keywords:** Microbiology, Diseases, Health care

## Abstract

This study aimed to assess the effect of smoking different tobacco types on the supragingival microbiome and its relation to dental caries. Forty supragingival plaque samples were collected from smokers of a single tobacco type and non-smokers seeking treatment at the University Dental Hospital Sharjah, UAE. DMFT (decayed, missing and filled teeth) was determined for all participants who were divided into two groups: no-low caries (NC-LC: DMFT = 0–4; n = 18) and moderate-high caries (MC-HC: DMFT = 5–20; n = 22). 16S rRNA gene was sequenced using third-generation sequencing with Nanopore technology. Microbiome composition and diversity were compared. Caries was most common among cigarette smokers. Supragingival microbiota were significantly altered among smokers of different tobacco types. In cigarette smokers, cariogenic bacteria from genus *Streptococcus* (including *S. mutans*) were significantly more among subjects with NC-LC, while* Lactobacilli* (including *L. fermentum*) were more among subjects with MC-HC. In medwakh smokers, several periodontopathogens were significantly elevated in subjects with NC-LC, while other pathogenic bacteria (as *Klebsiella pneumoniae*) were more in those with MC-HC. Cigarette and alternative tobacco smoking had a significant impact on the supragingival microbiome. Indeed, further studies are required to unravel the consequences of oral dysbiosis triggered by smoking. This could pave the way for microbiota-based interventional measures for restoring a healthy oral microbiome which could be a promising strategy to prevent dental caries.

## Introduction

Tobacco use is the leading preventable cause of mortality worldwide^1^. Globally, as well as in the Middle East, cigarette smoking has attracted serious public health concern. According to the WHO report on global tobacco use in 2019, the prevalence of active cigarette smoking among adults in the UAE was approximately 28% in men, but less in women^2^. Alternative forms of tobacco smoking such as shisha smoked in waterpipes are also common in the Middle East and spreading worldwide^3,4^. Dokha is another alternative form of tobacco consisting of tobacco mixed with herbs and other substances, smoked out of a narrow pipe called medwakh^5^. The Global Youth Tobacco Survey in 2013 demonstrated that the use of tobacco products in any form among youths aged 13–15 years in the UAE was 12.5%, and a higher percentage of young smokers used medwakh (9.1%) than cigarettes (6.2%)^6^.

The human oral cavity has one of the most complex and diverse microbial communities in the human body^7,8^. The oral microbiome plays key roles in human health and contributes to many diseases including both oral and systemic diseases such as cardiovascular disease^9^ and cancer^10^. For instance, high abundances of *Rothia* and *Neisseria* genera have been found essential for the maintenance of homeostasis and cardiovascular health, while high abundances of *Prevotella* and *Veillonella* genera are detrimental to homeostasis^11^. The composition of the oral microbiome is dependent upon environmental conditions^8^. Tobacco smoking affects the microbial ecology of the oral cavity through oxygen deprivation, antibiotic effects or other possible mechanisms leading to unbalanced microbiome or dysbiosis^12^. Loss of beneficial oral species due to smoking can lead to pathogen colonization and ultimately to progression of oral diseases such as periodontal disease and dental caries^11^.

Dental caries is one of the most prevalent chronic diseases, affecting approximately 2.4 billion people worldwide^13^. Dental caries can cause significant pain and negative overall quality of health. The burden of dental caries lasts for a lifetime because once the tooth structure is destroyed, it will need restoration and maintenance throughout life^14^. Dental caries is a multifactorial disease that initiates from microorganisms in the oral cavity and host factors^15^. Bacteria colonizing tooth surfaces in the form of supragingival dental plaque or biofilm are responsible for tooth decay. *Streptococcus mutans* was the first species isolated from caries lesions and has been considered the main cariogenic bacteria for decades^16^. Aetiological concepts of dental caries have evolved over the years from being caused by specific bacteria, to the current theory that highlights the critical changes in ecological stability resulting in caries development^17^.

In recent years, next-generation sequencing technologies have successfully been applied for oral microbial analysis^18,19^. It has been reported that bacterial diversity in the supragingival plaques of healthy individuals is different and higher than bacterial diversity in patients with dental caries, and this diversity gradually decreases with the severity of dental caries^20^. However, the diversity and structure of supragingival plaque microbiota in relation to smoking different types of tobacco and dental caries have not yet been investigated. In this study, it is the first time that tobacco use, microbiome, and dental caries have all been connected together. The aim of this study was to assess the effect of smoking different types of tobacco on the supragingival microbiome and its relation to dental caries in adults using third-generation sequencing with Nanopore technology.

## Material and methods

### Study population

Adult patients who sought dental treatment at the University Dental Hospital Sharjah, UAE, during the period of one year (October 2018– October 2019), were invited to participate in the study. Patients who fulfilled the inclusion criteria, agreed to participate in the study and signed the informed consent were recruited. Adults aged between 18 and 60 years who had at least 10 teeth in their mouth, being either non-smoker, or smoker of one type of tobacco only (cigarettes, shisha or medwakh only and no other tobacco product) were included in the study. Patients who were edentulous or had less than 10 teeth were excluded. Patients who received any periodontal treatment during the last 3 months, and patients who were on antibiotic or steroid therapy during or within 3 months of the study^21^, and those with ongoing orthodontic therapy were excluded. Medically compromised patients (patients with chronic systemic diseases or therapy other than cardiovascular diseases, diabetes, and obesity) and female patients who were pregnant were excluded. A total of 40 patients were included, four equal groups (n = 10 in each) including non-smokers and smokers of either cigarettes, shisha or medwakh. The study protocol was approved by the Research Ethics Committee at the University of Sharjah, UAE (REC-18-10-23-01), in compliance with the national and international standards including Helsinki declaration. All methods and procedures were performed in accordance with the relevant guidelines and regulations.

### Oral clinical examination and questionnaire

Information about participants’ general health status, detailed smoking history, and oral hygiene habits was obtained by a questionnaire. The oral clinical examinations were performed by a trained dental professional and consisted of a visual assessment of the oral mucosa, tongue, palate and floor of the mouth for any soft tissue abnormalities. The presence of dental plaque on tooth surfaces was recorded when clearly visible and expressed using the visible plaque index (VPI)^22^. The proportion of surfaces (%) with visible dental plaque was calculated for each subject. The sum of decayed, missing, and filled teeth (DMFT) was calculated according to the criteria from the World Health Organization^23^. DMFT describes the severity of dental caries in an individual and expresses dental caries experience until the day of examination^24,25^. In accordance with the DMFT index, the 40 subjects were divided into 2 groups as follows^20^;

1. No-caries to low-caries (NC-LC; DMFT = 0–4)
2. Moderate-caries to high-caries (MC-HC; DMFT =  > 4)

### Specimen collection

Supragingival plaques were collected from all participants using sterile Gracey curettes (Hu-Friedy, USA). Sample collection included all the supragingival plaque accumulated on the buccal surfaces of maxillary first molars and lingual surfaces of mandibular first molar teeth. However, if any of the molars were missing, we chose the mesial adjacent tooth after isolation with cotton rolls and air drying of the area to prevent contamination with saliva^20^. Samples were placed in 1.5 ml micro centrifuge tubes with 300 μl of sterile phosphate buffer. Samples were placed on dry ice and then transferred to − 80 °C freezer until further analysis.

For the molecular testing, we selected samples from the area with maximum plaque accumulation on the teeth. This was based on the quantity of DNA extracted from the plaques. From each subject, the sample with highest quantity of DNA (thus conforming maximum plaque accumulation) was used for sequencing.

### Sample processing and microbiome profiling

DNA extraction from each sample was done using Epicentre MasterPure™ DNA Purification Kit (Epicenter, USA), as recommended by the manufacturer. Assessment of the quality and quantity of the extracted DNA was done using a nanodrop (Colibri Microvolume Spectrometer; Titertek-Berthold, Germany). Amplification of the entire (~ 1500 bp) 16S rRNA gene was done using the 16S Barcoding Kit (SQK‐RAB204; Oxford Nanopore Technologies, Oxford, UK) and LongAmp™ Taq 2 × Master Mix (New England Biolabs, UK) with 1 µg of input DNA per sample. Purification of PCR products was done by using AMPure XP (Beckman Coulter, USA) followed by quantification by fluorometer Qubit 4 (Thermo Scientific, US). Equimolar amounts of the amplification products were pooled together, then a total of 100 ng DNA of the pooled sample was used for library preparation. Microbiota were identified using third-generation sequencing with Nanopore technology, on a MinION device (Oxford Nanopore Technologies, UK). MinION™ sequencing was performed using R9.4 flow cells (Oxford Nanopore Technologies, UK) according to the manufacturer's instructions. MinKNOW version 2.0 (Oxford Nanopore Technologies, UK) was used for live base calling and data acquisition. Raw data were converted into FASTQ format using Guppy v3.4.4, followed by demultiplexing, removal of nanopore and adaptor sequences. FASTQ files were analyzed on Nanopore EPI2ME platform with default minimum Q score of 7. Preliminary bacterial identification was done via ‘What’s in my Pot?’ (WIMP) workflow provided by Oxford Nanopore Technologies, UK. Reads assigned to all targets were re-analyzed by Kraken taxonomic sequence classification system (version 2.0.8-beta)^26^ using Partek® Genomics Suite® software, version 7.0 (Copyright ^©^ 2020; Partek Inc., St. Louis, MO, USA). The numbers of reads assigned per taxon were counted and the relative abundance of reads per taxon were used for separate downstream analysis, as described in our previous publication^27^.

### Statistical and bioinformatics analyses

Continuous variables were presented using mean ± SD. Clinical, demographic, microbiota relative abundance and alpha diversity were compared using the Kruskal–Wallis test for samples grouped based on tobacco types smoked. Correlations between relative abundance of taxa and DMFT were calculated using Spearman correlation coefficients (SPSS software, version 20). All statistical tests were two-sided. A P-value < 0.05 was considered statistically significant. Venn diagrams were generated to show the shared and unique Operational Taxonomic Units (OTUs) among groups, based on the occurrence of OTUs in a group regardless of their relative abundance using Venny bioinformatic tool (version 2.1)^28^. Shared taxa (genera and species) present in all four groups were defined as the core microbiome. For the species, relative abundance values were transformed into log2 values, then the mean value of each smoking group was normalized against the non-smokers as a control group to calculate log2 fold changes between non-smokers and smokers of each tobacco type independently for each bacterial taxa identified^27^. Heatmap was constructed using R version 4.0.1 (package: gplots; function: heatmap.2). Vegan package in R was used to calculate the estimates of alpha diversity (within sample diversity), including Shannon’s and Simpson’s diversity indices, estimators for richness including Chao1-type (diversity from abundance data), and Pielou’s index of species evenness. To compare alpha diversity metrics among groups, the non-parametric Kruskal–Wallis test or Mann–Whitney U test were utilized. For beta diversity analysis, dissimilarity matrix between samples was calculated with Bray Curtis index (abundance data; a measure of community composition differences between samples based on OTU counts, regardless of taxonomic assignment)^29^ and Jaccard index (presence absence data)^30^ using *vegdist* function from Vegan package in R. Based on the species level classification, principal component analysis (PCA) was conducted to evaluate the similarity of microbial community structure among all samples. PCA was done using “prcomp” function of package (ggfortify) in R.

## Results

### Clinical features of the study population

The majority of participants (85%) were males and ranged in age from 18 to 62 years. Eighteen (45%) participants had no or low (NC-LC) caries (DMFT = 0–4) and 22 (55%) participants had moderate-high (MC-HC) caries (DMFT = 5–20). Within smoking groups, it was found that dental caries (MC-HC) was more common among cigarettes smokers (n = 8; 80%), followed by shisha smokers (n = 6; 60%), medwakh smokers (n = 5; 50%) and non-smokers (n = 3; 3%).

Visible Plaque Index (VPI) was not significantly different among different smoking groups (P > 0.05), although smokers of cigarettes had the highest VPI (mean ± SD = 0.50 ± 0.30) compared to medwakh smokers (0.38 ± 0.25), shisha smokers (0.37 ± 0.18) and non-smokers (0.30 ± 0.26). DMFT was not significantly different among different smoking groups (P > 0.05), although smokers of cigarettes had the highest DMFT (9.7 ± 6.0) compared to medwakh smokers (5.60 ± 3.60), shisha smokers (5.80 ± 4.26) and non-smokers (4.30 ± 4.90).

Regardless of smoking, VPI was not significantly different between participants who had NC-LC and MC-HC (mean ± SD = 0.37 ± 0.26 and 0.40 ± 0.25, respectively), but DMFT was significantly different between these two groups (2.11 ± 1.45 in NC-LC and 9.82 ± 0.14 in MC-HC). Both VPI and DMFT were not significantly different among smokers of different tobacco types in each of the NC-LC and MC-HC groups. There was no significant correlation between DMFT and VPI (P > 0.05); however, there was significant positive correlation between DMFT and duration of smoking (correlation coefficient = 0.375; P = 0.017). Clinical data of all participants is included in Supplementary data file 1.

### Bacterial abundance and distribution

#### Phyla

*Firmicutes* was the most abundant phylum in the supragingival plaque samples collected from non-smokers, smokers of cigarettes, medwakh and shisha. Abundance of *Proteobacteria* was significantly greater in shisha smokers compared to medwakh smokers. *Actinobacteria* were significantly more present in shisha smokers compared to cigarette smokers.

**Figure 1:**
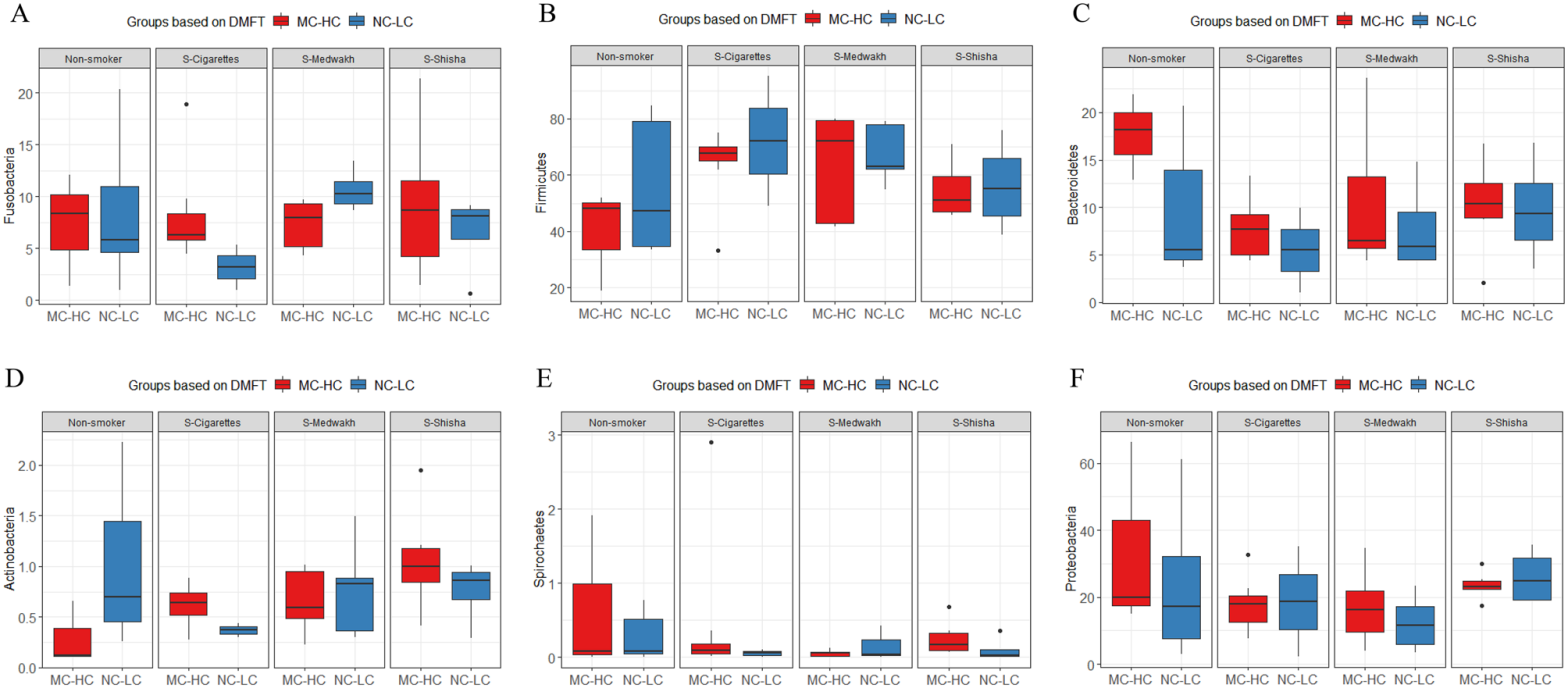
Comparison of the relative abundance (%) of the major phyla detected in the supragingival plaque samples; collected from subjects with no or low (NC-LC) caries and moderate-high (MC-HC) caries, in smokers of different types of tobacco and non-smokers. Box plots show Q1-median-Q3 with data range. Black dots are outlier values.

Comparison of the relative abundances of phyla detected in the 40 supragingival plaque samples from smokers of different types of tobacco and non-smokers in relation to dental caries status are shown in Fig. 1. *Fusobacteria* was found significantly more often in medwakh than shisha smokers in patients with no-low caries. For those in the moderate-high caries group, *Spirochaetes* was found significantly less often in medwakh than shisha smokers. *Actinobacteria* was found significantly more often in shisha smokers than in cigarettes smokers and non-smokers. *Bacteroidetes* were significantly more common in non-smokers than shisha and cigarette smokers and *Firmicutes* were significantly more common in non-smokers than cigarette smokers*.*

**Figure 2:**
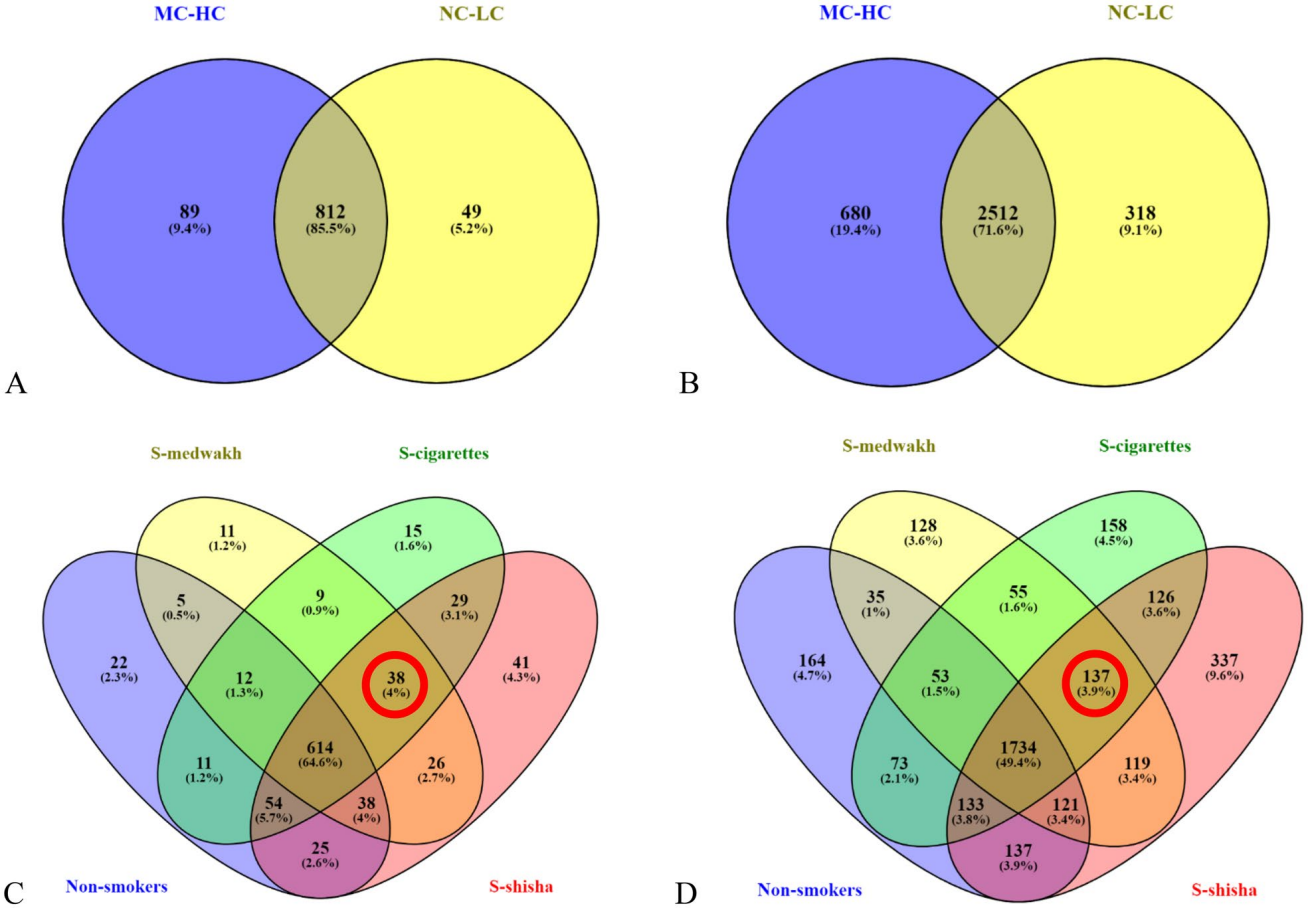
Venn diagram of exclusive and shared taxonomical unique microbiota (**A** and **C**: genus, and **B** and **D**: species) detected in supragingival plaque samples from patients with no-low caries and moderate-high caries (**A**,**B**), non-smokers and smokers of cigarettes, medwakh and shisha (**C**,**D**). Red circled numbers represent genera and species detected exclusively in smokers.

Supplementary Tables 2 and Fig. 1 show the relative abundance of phyla detected in supragingival plaque samples from the 4 study groups (i.e. non-smokers, cigarettes, medwakh and shisha). Additionally, we compared the number of genera and species detected in the samples (grouped based on either smoking status or dental caries). In subjects with MC-HC, the number of genera (n = 901) and species (n = 3192) detected in at least one sample was higher than those with NC-LC (n = 861 and 2830, respectively). Figure 2 shows the number of common genera (A and C) and species (B and D) in both groups and those found exclusively in one group. The number of genera and species exclusively found in subjects with MC-HC was almost double those found in subjects with NC-LC. As for smoking, 614 (64.6%) genera and 1743 (49.4%) species were shared between all the groups. Shisha smokers had the highest number of exclusive genera and species whereas medwakh smokers had the least (Fig. 2).

#### Genera

**Figure 3:**
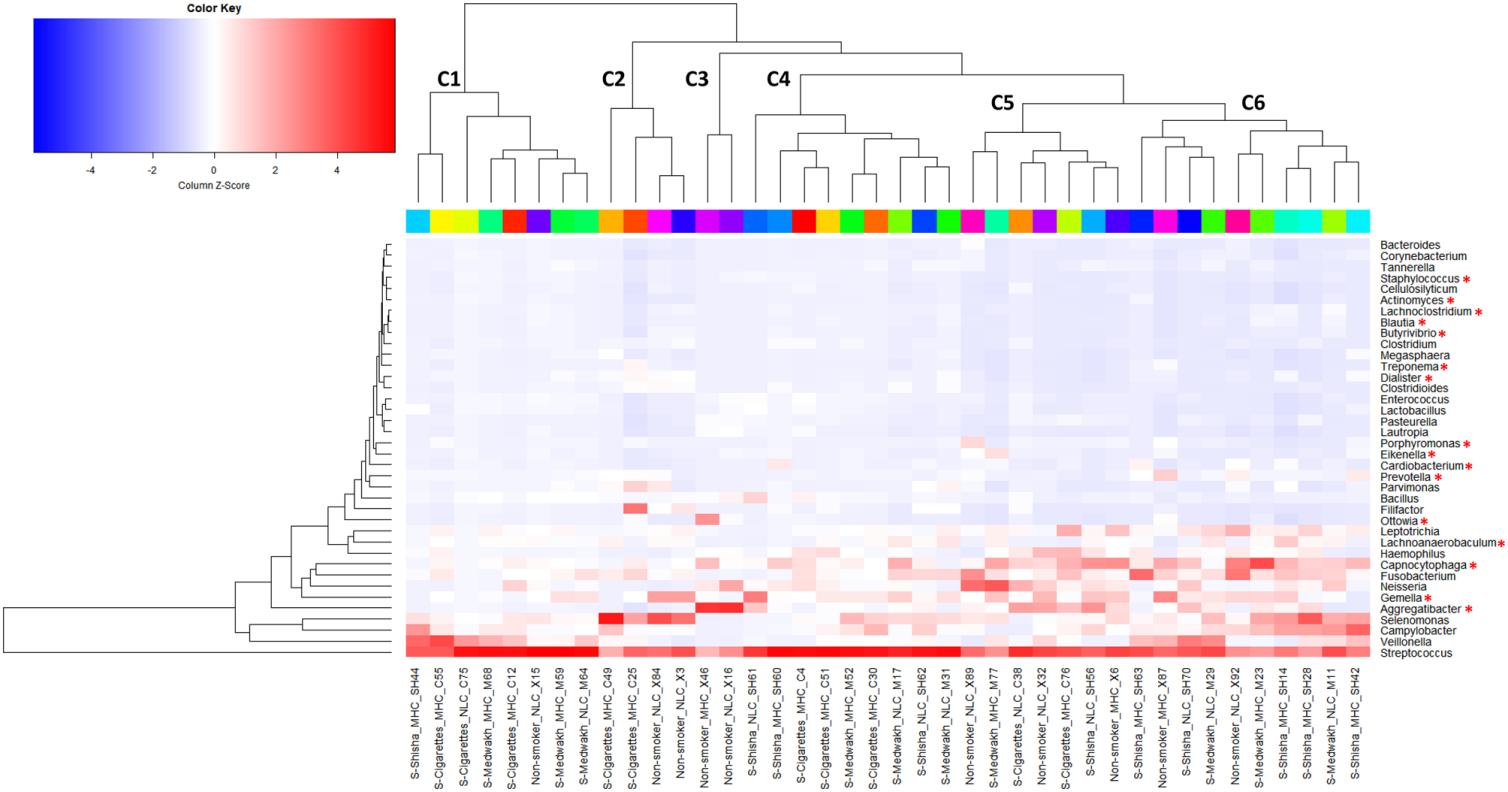
Distribution of different genera (relative abundance ≥ 1%) in the 40 supragingival plaque samples. Smoking status and caries are included in each sample’s label. Red color indicates high abundance and blue color indicates low abundance of each bacterial genera. Dendrograms show clustering based on the relative abundance of different genera (left dendrogram: genera clustering; top dendrogram: clustering of samples based on abundances of different genera). 16 genera were significantly different between the study groups (red asterix). NLC: no-caries to low-caries; MHC: moderate-caries to high-caries.

For statistical analyses, genera with relative abundance of ≥ 1% in at least one sample were investigated (shown in Fig. 3). Based on the relative abundance of genera, the samples were differentiated into 6 clusters (C1-C6 in Fig. 3). Each cluster contained samples from various smoking groups with NC-LC or MC-HC, and no cluster contained any specific group exclusively.

**Figure 4:**
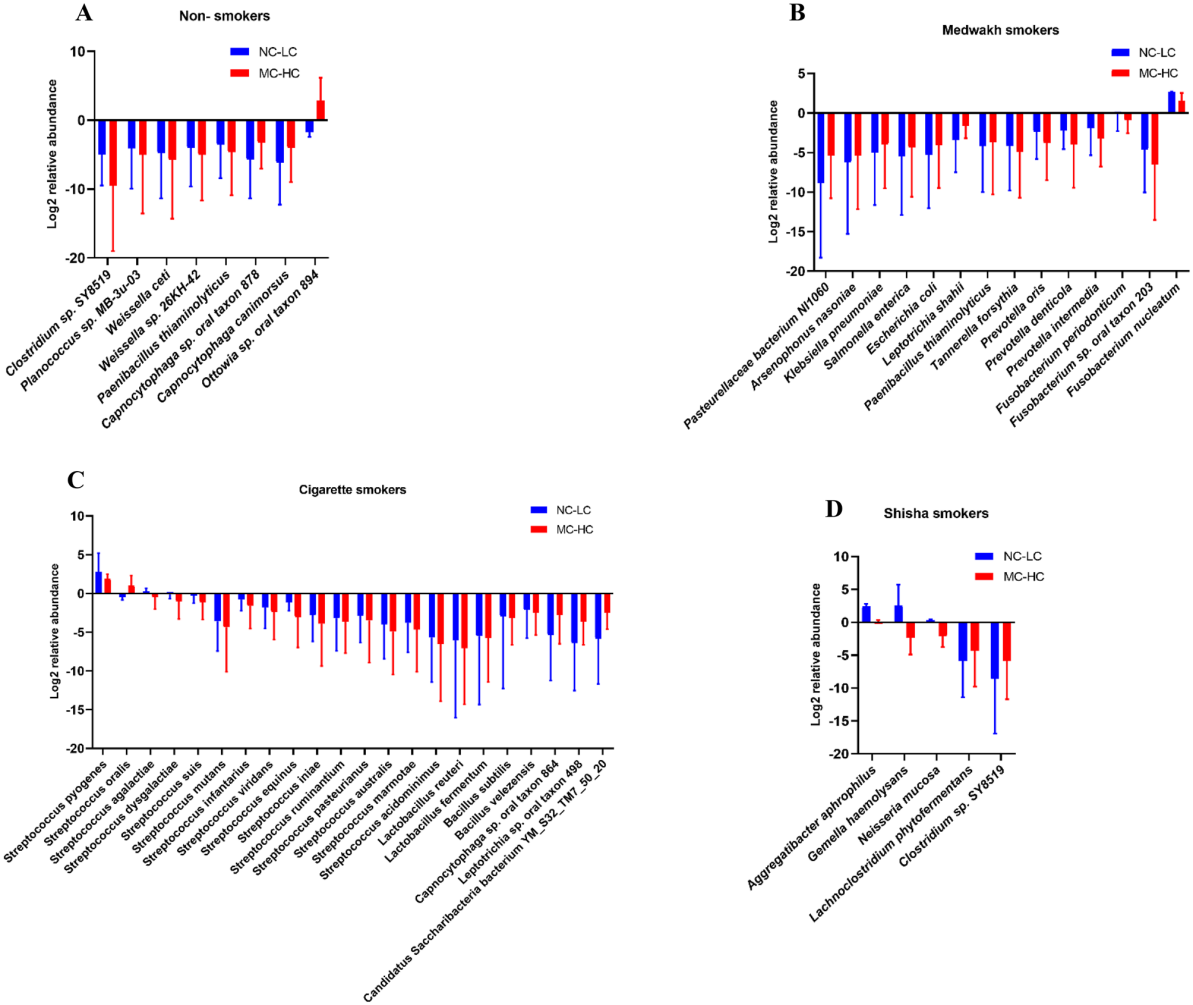
Comparison of the relative abundance (%) of species detected in the supragingival plaque samples collected from patients with no or low (NC-LC) caries and moderate-high (MC-HC) caries, in smokers of different types of tobacco (3B, 3C, 3D) and non-smokers (3A).

We compared the relative abundance of the major genera detected in the supragingival plaque samples collected from patients with NC-LC and MC-HC, in smokers of different types of tobacco and non-smokers (shown in Fig. 4).

Overall, the most abundant genus in this study was *Streptococcus* (27.3% of the total abundance), which was also the most abundant genus in each of the 40 samples tested. Other common genera are *Selenomonas* (7.2%), *Capnocytophaga* (6.3%), *Veillonella* (6.2%), *Gemella* (5.6%), *Campylobacter* (5.2%), *Fusobacterium* (5.2%), *Aggregatibacter* (4.7%) and *Neisseria* (4.3%). A total of 16 genera were significantly different between the study groups considering both smoking and dental caries (marked with red asterix in Fig. 3, also shown in Supplementary Fig. 2).

Most of the alterations were seen in the MC-HC group, especially among smokers. *Ottowia* was significantly increased and *Actinomyces* was significantly decreased in non-smokers than in smokers of any tobacco type (medwakh, shisha or cigarettes) with MC-HC. *Capnocytophaga* and *Aggregatibacter* in cigarette smokers were significantly decreased compared to non-smokers with MC-HC. *Cardiobacterium* was also decreased in cigarettes smokers compared to both shisha and medwakh smokers with MC-HC. *Gemella* was significantly increased, and *Treponema* was significantly decreased in medwakh smokers than shisha smokers with MC-HC. Compared to non-smokers, *Staphylococcus* was significantly greater and *Porphyromonas* was significantly less in medwakh smokers with MC-HC.

For participants with NC-LC, *Butyrivibrio* and *Dialister* were significantly increased in non-smokers compared to shisha smokers. *Eikenella* was significantly increased and *Lachnoanaerobaculum* was significantly decreased in non-smokers compared to medwakh smokers. *Blautia, Lachnoclostridium* and *Prevotella* were significantly more and *Aggregatibacter* was significantly less in medwakh smokers than shisha smokers with NC-LC (all are shown in Supplementary Fig. 2).

#### Species

Species (n = 271) with relative abundance > 0.1% in at least one sample were considered in the analysis. A list of all species detected in each sample was included in Supplementary Table 3, which shows the relative abundance (%) of all species detected in the supragingival plaque samples from non-smokers, smokers of cigarettes, medwakh and shisha.

Overall, a few species (n = 5) were significantly different between patients with no-low and moderate-high caries (regardless of smoking status), namely *Campylobacter gracilis*, *Capnocytophaga sp. oral taxon 864*, *Leptotrichia sp. oral taxon 498*, *Weissella sp. 26KH-42*, *Candidatus Gracilibacteria bacterium HOT-871.* However, more significant differences were found within each smoking group, when subjects with NC-LC and MC-HC were compared for species in the supragingival samples. In non-smokers, significant differences were found in 8 species (shown in Fig. 4A); however, none of them were related to dental caries. In medwakh smokers, several periodontopathogens (*Fusobacterium nucleatum, Fusobacterium periodonticum, Prevotella intermedia, Prevotella denticola, Tannerella forsythia*) were significantly elevated in patients with no-low caries compared to moderate-high caries. Other pathogenic bacteria such as *Klebsiella pneumoniae, Escherichia coli* and *Salmonella enterica* were significantly increased in patients with moderate-high caries compared to no-low caries (shown in Fig. 4B). Except for *Streptococcus oralis,* other members of the genus *Streptococcus* (*S. pyogenes, S. agalactiae, S. dysgalactiae, S. suis, S. mutans, S. infantarius, S. viridans, S. iniae, S. ruminantium, S. pasteurianus, S. australis, S. acidominimus*, *S. equinus* and *S.marmotae*) were significantly more among cigarette smokers with no-low caries compared to moderate-high caries. On the other hand, *Lactobacilli* including *Lactobacillus reuteri* and *Lactobacillus fermentum* were significantly increased among cigarette smokers with moderate-high caries compared to those with no-low caries (shown in Fig. 4C). For shisha smokers, significant differences were found in 5 species (shown in Fig. 4D); however, none of them were related to dental caries. Important pathogens such as *Aggregatibacter aphrophilus* and *Gemella haemolysans* were significantly elevated in shisha smokers with no-low caries compared to those with moderate-high caries (shown in Fig. 4D).

**Figure 5:**
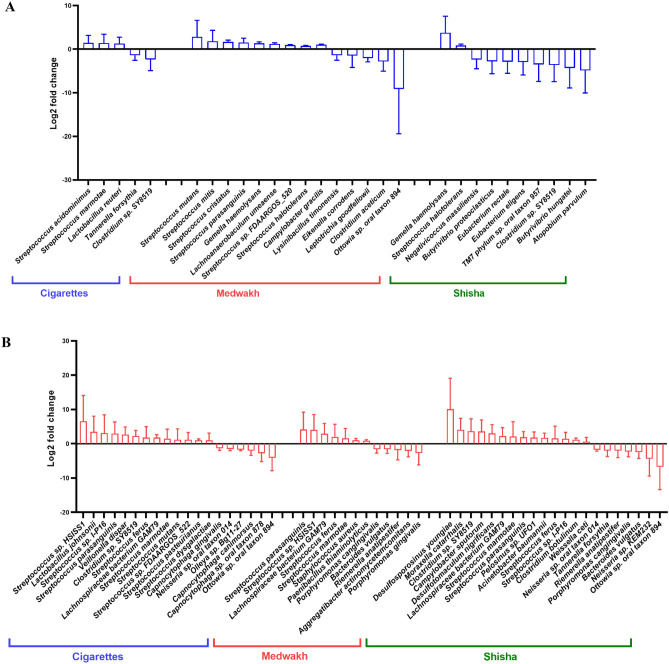
Species significantly altered in smokers of different tobacco types. Data represent Log2 folds change of the relative abundance of each species in smokers compared to non-smokers (**A**) in no-low caries group (**B**) in moderate-high caries group.

Smokers of different tobacco types were compared to non-smokers in both NC-LC and MC-HC groups (Fig. 5A,B). In patients with no-low caries, we found significant difference in the relative abundance of multiple species in smokers of different tobacco types compared to non-smokers (shown in Fig. 5A). Interestingly, smokers had significantly higher abundance of multiple species from the genus *Streptococcus*, namely *S. marmotae* and *S. acidominimus* in cigarette smokers*, S. mutans, S. cristatus, S. mitis, S. parasanguinis, S. halotolerans and S. sp. FDAARGOS_520* in medwakh smokers, and *S. halotolerans* in shisha smokers. For cigarette smokers, *Clostridium sp. SY8519* and *Tannerella forsythia* were significantly increased compared to non-smokers, while other species were decreased (shown in Fig. 5A).

In patients with moderate-high caries, we found significant differences in the relative abundance of multiple species in smokers of different tobacco types compared to non-smokers (shown in Fig. 5B). Interestingly, smokers had significantly higher abundance of multiple species from the genus *Streptococcus*, namely *S. parasanguinis, S. pasteurianus, S. dysgalactiae, S. marmotae, S. mutans, S. sp. FDAARGOS_522, S. ferus, and S. sp. I-P16* in cigarette smokers, *S. parasanguinis, S. ferus, S. marmotae and S. sp. HSISS1* in medwakh smokers, and *S. parasanguinis, S. ferus, S. marmotae, and S. sp. I-P16* in shisha smokers. For medwakh smokers, several periodontopathogens such as *Porphyromonas gingivalis, Porphyromonas cangingivalis and Aggregatibacter actinomycetemcomitans* were significantly decreased. Pathogenic bacteria such as *Moraxella catarrhalis* and *Acinetobacter baumannii* were significantly elevated in shisha smokers compared to non-smokers.

**Figure 6:**
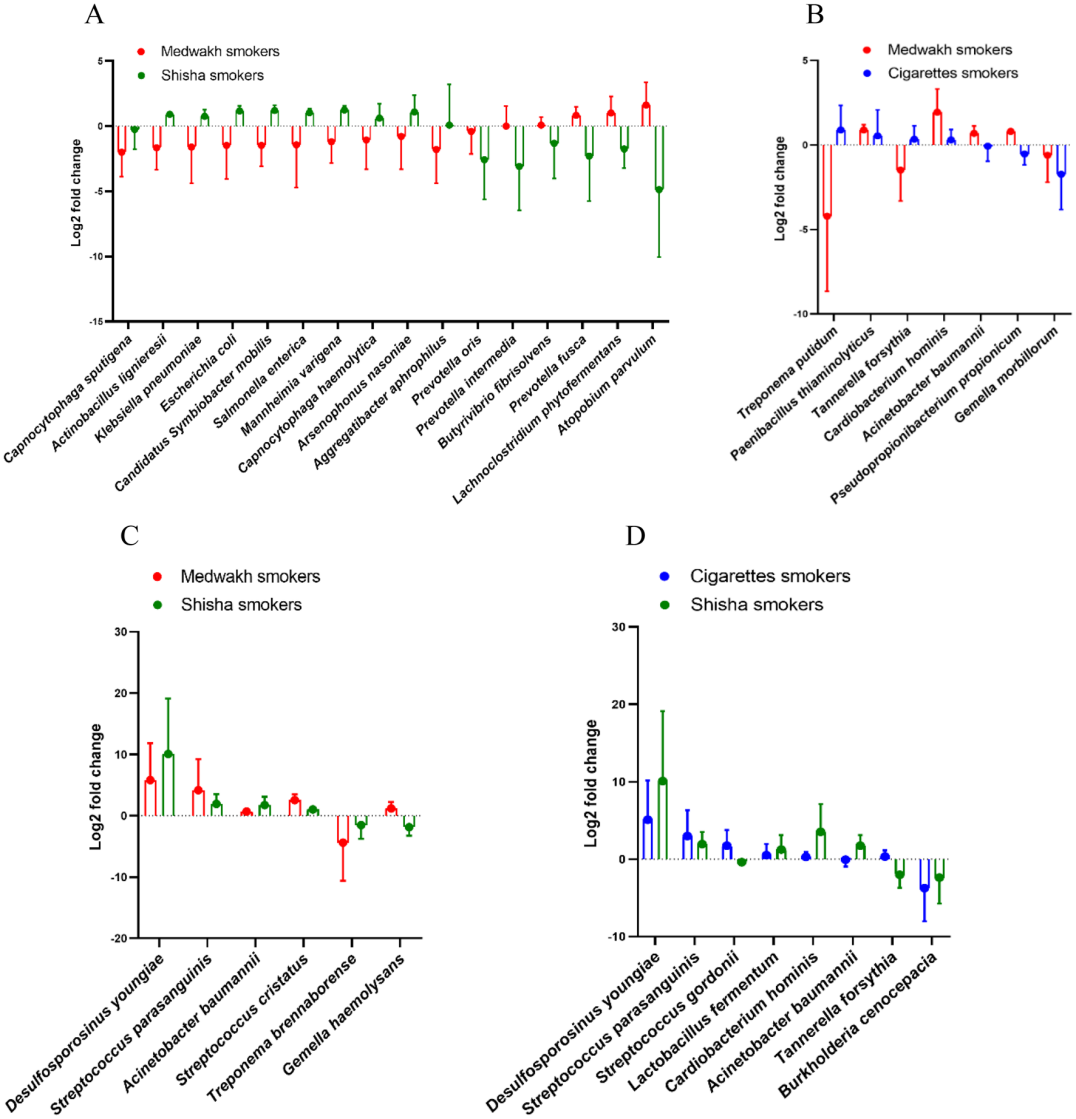
Comparison of the relative abundance of species detected in smokers of different tobacco types in patients with no-low caries (**A**) and patients with moderate-high caries (**B**–**D**). Log2 folds change of the relative abundance of each species in smokers compared to non-smokers are shown. Species with significant difference in the relative abundance were shown.

Intergroup comparisons of smokers of different tobacco types revealed non-significant differences in the relative abundance of different species in cigarette smokers compared to smokers of shisha and medwakh with no-low caries. However, the latter two groups showed significant differences in the relative abundance of 16 species (shown in Fig. 6A). There was a significant increase in several pathogenic organisms such as *Klebsiella pneumoniae, Escherichia coli,* and *Salmonella enterica* in the samples collected from shisha smokers in the no-low caries group. An important periodontopathogen (*Prevotella intermedia*) was significantly increased in medwakh smokers, compared to shisha smokers with no-low caries.

Intergroup comparisons in the moderate-high caries group revealed the presence of significant differences among smokers of different tobacco types (Fig. 6B–D). A total of 7 species were significantly different in cigarette smokers compared to medwakh smokers. An important periodontopathogen (*Tannerella forsythia*) was significantly decreased in medwakh than cigarette smokers (Fig. 6B). A total of 6 and 7 species were significantly different in shisha smokers compared to medwakh and cigarette smokers, respectively (Fig. 6C,D). *Streptococcus parasanguinis* was significantly decreased in shisha smokers compared to both medwakh and cigarette smokers, *Streptococcus gordonii* and *Tannerella forsythia* were significantly decreased in shisha smokers compared to cigarette smokers, while most of the other species were elevated when compared to medwakh and cigarette smokers.

### Bacterial diversity

**Figure 7:**
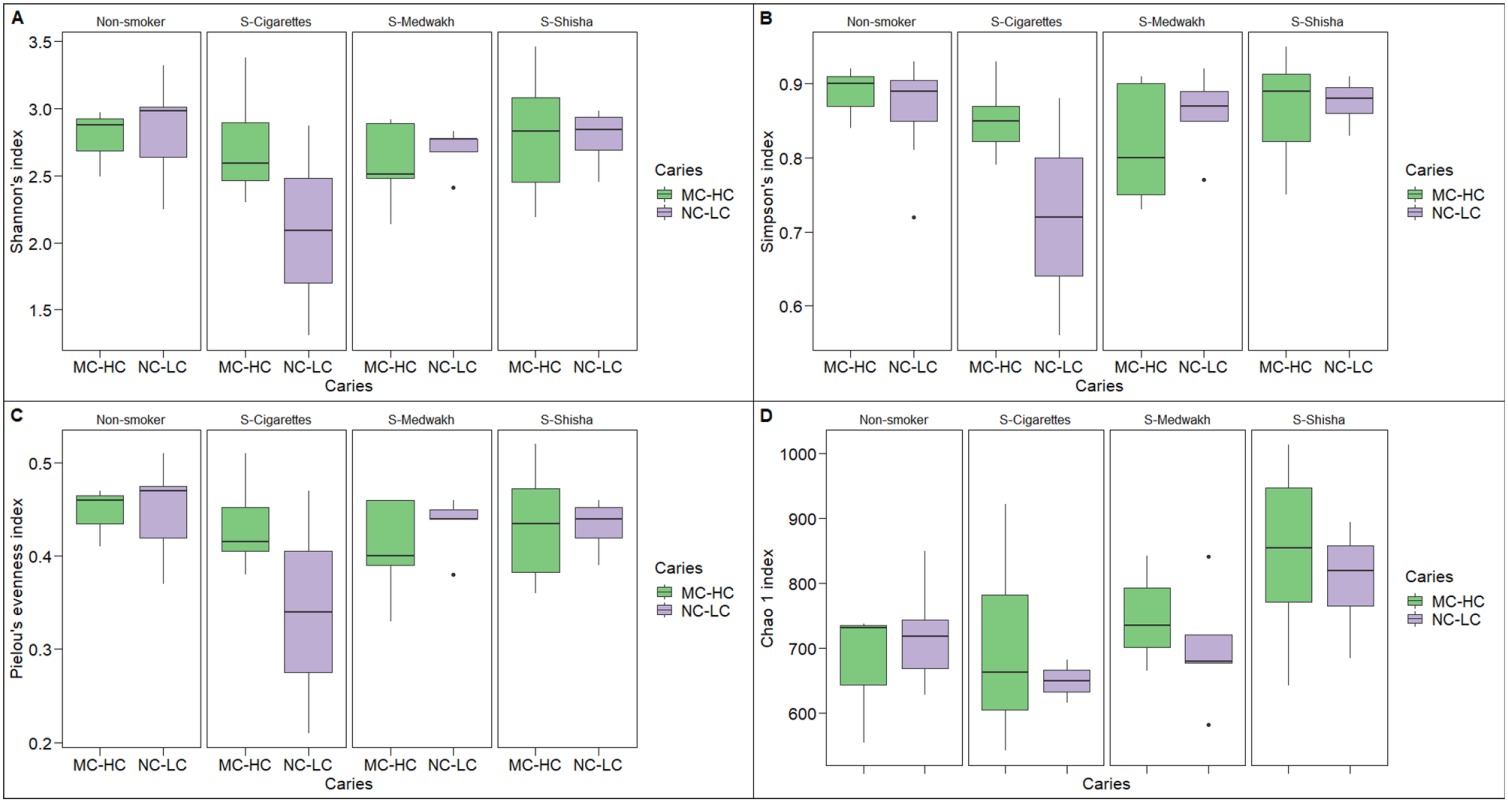
Alpha diversity in the 40 supragingival plaque samples grouped based on type of tobacco smoking and dental caries. Shannon’s (**A**) and Simpson’s (**B**) diversity indices, Pielou’s index (**C**) for species evenness and Chao1 index (**D**) for richness are shown. Box plots show Q1-median-Q3 with data range. Black dots are outlier values.

The alpha diversity indices of Shannon, Simpson, Chao1 and Pielou’s index evenness are shown in Fig. 7. There was no statistically significant difference between the study groups for both caries and smoking types (P > 0.05). Although not statistically significant, the Shannon, Simpson and Pielou’s values for supragingival plaque samples from cigarette smokers with NC-LC were lower than those with MC-HC, as well as other groups of smokers and non-smokers (Fig. 7 A-C). As for Chao1 index, it was higher in shisha smokers with both NC-LC and MC-HC compared to other groups of smokers and non-smokers. (Fig. 7D).

**Figure 8:**
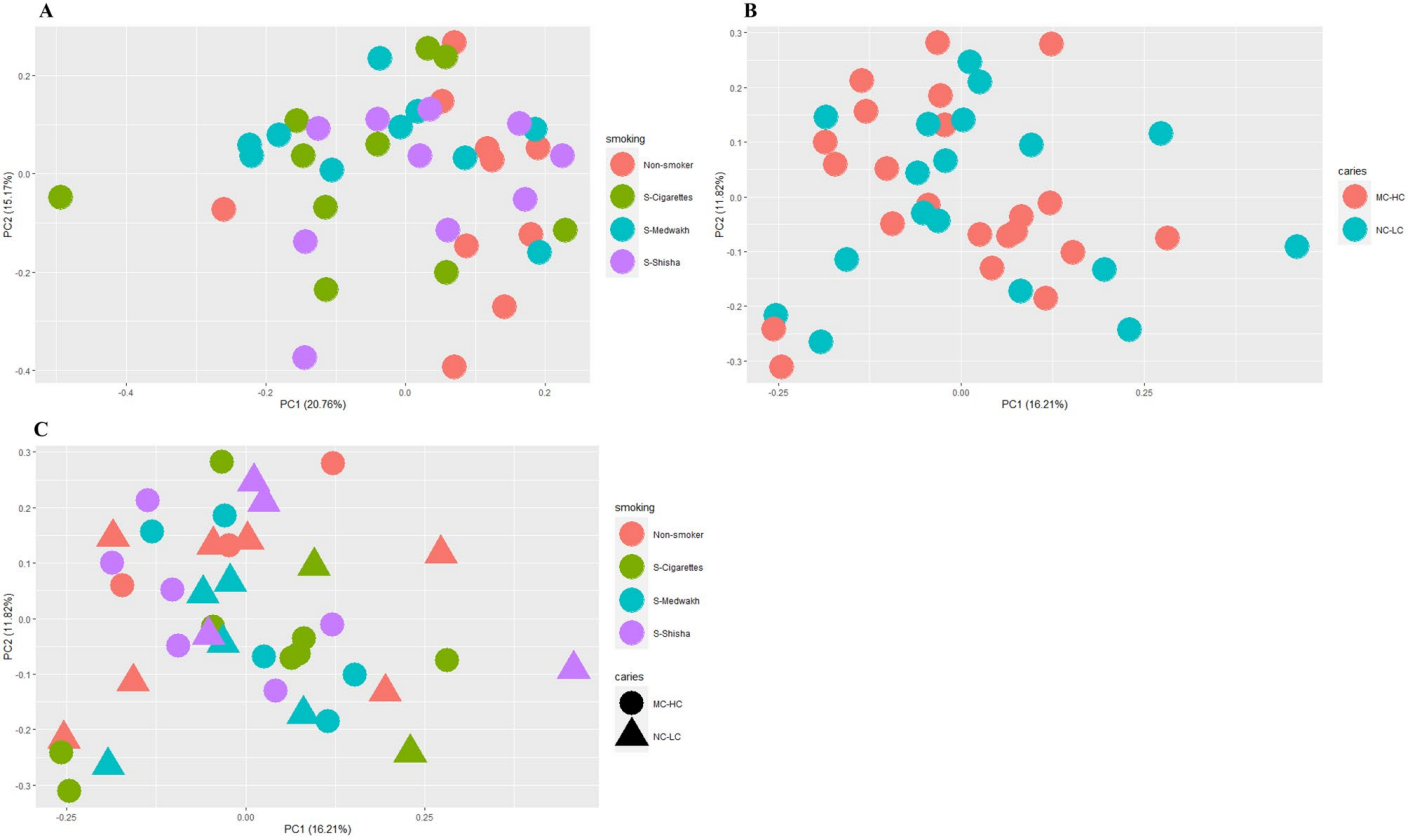
Principal component analysis of the 40 supragingival plaque samples grouped based on type of tobacco smoking (**A**) and dental caries (**B**). Each dot represents a microbial community from one sample. Samples grouped based on both tobacco smoking type and dental caries are shown in (**C**), in which; tringles represent the NC-LC samples and circles represent the MC-HC samples.

Our results indicate that similar community structures of supragingival plaques were present in the study groups (based on tobacco smoking and dental caries) according to PCoA analysis and Bray–Curtis and Jaccard PCoA plots (Fig. 8 and Supplementary Fig. 3). As seen in the figures, microbial communities from smokers of different tobacco types and also caries groups overlapped; no distinct clusters were identified.

## Discussion

It is well known that changes in the bacterial composition of dental plaques instigate dental caries^31^. There are many factors that contribute to these changes leading to caries. Earlier studies have demonstrated that exposure to nicotine promotes the cariogenic activity of oral microorganisms and the formation of a caries-susceptible environment^32^.

In this study, we found that the prevalence of dental caries (MC-HC) was increased among smokers, especially those using cigarettes. This is in agreement with a recent systematic review and meta-analysis of related studies that indicated a positive association between tobacco smoking and dental caries and a higher prevalence of caries among smokers compared to non-smokers^33^. We found that the VPI and DMFT, which are important risk indicators related to caries^34^, were not significantly different among smokers of different tobacco types. However, DMFT was positively correlated with the duration of smoking. A previous study revealed that long-term cigarette smoking is associated with multiple dental disorders including caries, but a cause-and-effect relationship is still not proven in this study or others^35^. It is possible that longer exposure to tobacco may predispose individuals to caries by inducing more profound alteration in the microbiota leading to oral dysbiosis^32^. In dysbiosis, the delicate equilibrium of the oral ecosystem is disrupted, allowing disease-causing pathogenic bacteria to manifest and predisposing individuals to various conditions such as caries^36^.

It has been shown that smoking has an impact on various stages of dental plaque formation. However, previous studies focused on investigating the effect of smoking on oral microbiome using either mouth wash or buccal swabs, which are not specific to caries. In contrast, we used supragingival plaque microbes that are directly attached to the tooth surface^37,38^. In our study, we explored the effects of different types of tobacco smoking on the supragingival plaque microbiome and its association with dental caries by specifically collecting isolated supragingival plaques from all participants using sterilized curettes. We believe that such an approach is more useful to reveal microbial alterations in smokers, as supragingival plaque is directly exposed to the tobacco smoked due to its location over the tooth surface. This approach was also used by Xiao, et al.^20^ and He, et al.^39^ who have investigated microbial changes in the supragingival plaques in adults with and without dental caries. Our results indicate that smoking different tobacco types is associated with different alterations in the supragingival microbiome at different taxonomic levels (phyla to species). These alterations are also linked to the stage of dental caries.

Although *Firmicutes* was the most abundant phyla in all participants; *Proteobacteria* and *Actinobacteria* were significantly elevated in shisha compared to medwakh and cigarette smokers, respectively. In a study investigating the microbiota in oral rinse specimens of smokers of different tobacco types in the USA, *Proteobacteria* was less abundant in the microbiome of cigarette smokers^37^. According to the latter study, other phyla including *Actinobacteria, Firmicutes,* and *Proteobacteria* were found more abundant in alternative tobacco smokers (e-cigarettes, hookah or shisha, and/or cigar/cigarillo) while *Bacteroidetes* and *Saccharibacteria* were more depleted. In those who only smoked shisha, genera *Porphyromonas, Leptotrichia, Streptobacillus, Fusobacterium*, and *Saccharibacteria* were depleted. One major limitation of the latter study is that the status of the oral health of the participants was not considered; thus, it is difficult to conclude that microbiota alterations were caused by tobacco use exclusively.

In our study, we considered both dental caries and smoking in our participants and we found variations in the microbiota related to the effect of both factors. For example, *Bacteroidetes* were significantly elevated in non-smokers compared to shisha and cigarette smokers, and *Firmicutes* were significantly increased in non-smokers than cigarette smokers*.* Additionally, both *Bacteroidetes* and *Firmicutes* were depleted in smokers with moderate-high caries; thus, both factors seem to play a role in the microbiota alteration. Compared to other studies, Xiao, et al. reported multiple dominant phyla in the plaque microbiota such as *Bacteroidetes, Actinobacteria, Proteobacteria, Firmicutes, Fusobacteria,* and TM7 that were not significantly different in subjects with or without caries^20^. It is noteworthy that the latter study used pyrosequencing to detect the microbiota in the samples, and other factors (such as smoking) affecting the microbiota composition were not considered. He et al. had also investigated the supragingival plaque microbiota in caries and healthy adults, and found that multiple phyla (*Proteobacteria, Firmicutes, Actinobacteria, Bacteroidetes, Fusobacteria,* and *Spirochaetes*) were dominant in both groups. Among these phyla, the relative abundance of *Bacteroidetes* and *Spirochaetes* were significantly higher in the caries patients than in the healthy individuals^39^. This is consistent with our findings showing that *Bacteroidetes* were depleted in subjects with moderate-high caries among smokers of different tobacco types. One major limitation of the He et al. study is that smoking was not considered; thus, it is not clear if microbial alteration in the latter study was exclusively caused by or associated with caries or attributed to other factors.

Furthermore, we investigated in depth both genera and species present in the samples. We found that the number of genera and species exclusively present in subjects with MC-HC was almost double those found in NC-LC subjects. This may indicate that subjects with MC-HC have more complicated microbiome as a consequence of dysbiosis in dental caries^40^. It is highly accepted that polymicrobial synergy of multiple microorganisms is a major contributor to the formation of a cariogenic biofilm, in which microbial community changes lead to caries progression^40^.

At the genus level, *Streptococcus* was the most abundant genera (27.3%) in this study, while another 16 genera were significantly different between the study groups. Most of the alterations were detected in MC-HC group, especially among smokers. Other studies have reported significant variation at the genus level between caries and healthy individuals. *Selenomonas, Treponema*, and *Atopobium* showed higher relative abundance in the caries group, whereas the *Bergeriella* had a higher relative abundance in healthy subjects^39^. In our study, *Treponema* was significantly less abundant in medwakh smokers than shisha smokers with MC-HC, and more genera were altered in relation to both smoking and caries.

Members of the genera *Streptococcus, Actinomyces, Neisseria, Veillonella, Capnocytophaga, Leptotrichia, and Haemophilus* are collectively known as early colonizing bacteria. On the other hand, later colonizing bacteria, such as members of the *Fusobacterium, Treponema, Tannerella, Prevotella, and Porphyromonas* genera, use adhesion receptors provided by the early colonizers^41^. In our study, we noticed that the genera that were altered in early stages of caries (NC-LC) were different from those altered at later stages (MC-HC). From the early colonizers, significant variations of some genera were seen only in subjects with MC-HC. *Actinomyces* was significantly more abundant in smokers of any tobacco type (medwakh, shisha and cigarettes) compared to non-smokers with MC-HC, while *Capnocytophaga* in cigarette smokers was significantly less abundant than in non-smokers with MC-HC. For the late colonizers, most of the significant variations were observed in medwakh and shisha smokers. *Treponema* was significantly more abundant in shisha smokers than medwakh smokers with MC-HC, while *Prevotella* was significantly more abundant in medwakh smokers than shisha smokers with NC-LC. *Porphyromonas* was significantly less abundant in medwakh smokers compared to non-smokers with MC-HC. These alterations confirmed that different tobacco types have different effects on the bacteria composing the oral biofilm at different stages of biofilm development, potentially predisposing individuals to dental caries, which is a polymicrobial disease caused by various bacterial consortia.

For decades, acid-producing bacteria, mainly Mutans *streptococci* (especially *Streptococcus mutans*) and *lactobacilli*, have been considered the main pathogenic bacteria of caries. However, more recent molecular analyses have revealed the existence of a pathogenic community that includes non-streptococcal bacteria such as *Bifidobacterium* and *Actinomyces*^40^. The effects of nicotine on different stages of caries are very well documented^42,43^. In the biofilm maturation stage, nicotine causes ecologic imbalance by encouraging *Streptococcus mutans, Lactobacilli, Streptococcus gordonii, Actinomyces aggregation* and facilitating *S. mutans* to compete with *S. sanguinis*^44^. An interesting finding in this study is that cigarette smokers in the NC-LC group had significantly more *streptococci* (including the cariogenic bacteria *S. mutans*) compared to those in the MC-HC group. On the other hand, *Lactobacilli* including *Lactobacillus reuteri* and *Lactobacillus fermentum* (also cariogenic bacteria) were significantly more abundant among cigarette smokers with moderate-high caries compared to those with no-low caries. This finding is in agreement with the hypothesis that the etiology of dental caries is community driven rather than the product of a single organism, and that *S. mutans* is not the sole microbe responsible for carious lesions^45^. This also highlights that the effect of cigarettes smoking on the microbiota is related to the stage of dental caries.

As for cigarettes, the effect of medwakh smoking on the microbiota was different in subjects at different stages of dental caries. Several periodontopathogens were significantly more abundant in medwakh smokers with NC-LC compared to MC-HC. Pathogenic bacteria such as *Klebsiella pneumoniae, Escherichia coli* and *Salmonella enterica* were significantly more abundant in medwakh smokers with MC-HC compared NC-LC. This is in accordance with the dynamic stability of the oral microbiota hypothesis where prolonged changes in microbial metabolic and loss of resilience factors may shift the ecological balance of the microbiota into either acidogenic or alkalinogenic stages, depending on the nature of the predominant substrates, leading to clinical diseases^46^.

For shisha smokers, significant differences were found in 5 species; however, none of them were related to dental caries. There was a significant increase in several pathogenic organisms such as *Klebsiella pneumoniae, Escherichia coli,* and *Salmonella enterica* in the samples collected from shisha smokers in the no-low caries group. It is possible that smoking equipment were contaminated with pathogenic bacteria, which might be a possible scenario if the subject was smoking them at public places where poor hygienic measures were followed^47^. If these pathogenic bacteria were introduced into the oral cavity, they can grow as the oral environment is supportive for their growth due to the toxic effect of tobacco^48^. The same explanation may be applicable to medwakh smokers if the smoking pipe was contaminated, as pathogenic bacteria were significantly more abundant in some medwakh smokers with MC-HC. Important pathogens such as *Aggregatibacter aphrophilus* and *Gemella haemolysans* were significantly more abundant in shisha smokers with NC-LC compared to those with MC-HC. Our findings are in agreement with a report from the UAE showing alterations in the oral microbiome of cigarette and medwakh smokers, but no effect on shisha smokers^38^. In our study, findings concerning shisha were not comparable to the latter report, considering that the latter report was based on testing the microbial composition of mouth rinse, which is not representative of the supragingival plaque microbiome investigated in our study.

For alpha diversity, no statistically significant differences were detected between the groups. This is in agreement with a previous report comparing the supragingival microbiome in subjects with and without caries in adults from China^39^. The latter study showed that the supragingival plaque of caries patients had higher evenness of species compared to healthy subjects. We could not find the same; however, we noticed that smokers of cigarettes at early stages of caries (NC-LC) had lower evenness (measured by Pielou’s index) than those with MC-HC. This was not shown in users of other tobacco types or non-smokers. We noticed that species richness (measured by Chao1 index) was greater in shisha smokers regardless of caries stages. It is noteworthy that shisha smokers had the highest number of exclusive genera and species compared to other groups (Fig. 2), which may explain the observed highest richness in shisha smokers. Another study compared between healthy subjects and those with caries of various severity, using similar criteria to those we used in grouping subjects according to caries severity, but without considering the smoking status. In the latter study, microbial diversity in healthy plaques exceeded that of dental caries, with the diversity decreasing gradually with the severity of caries^20^. We could not find the same; however, we noticed that smokers of cigarettes at early stages of caries (NC-LC) had lower species diversity (measured by Shannon and Simpson indices) than those with MC-HC. This was not shown in users of other tobacco types or non-smokers. This finding confirms the effect of cigarette smoking on the supragingival microbiota, and smoking’s ability to reduce microbial diversity, even in subjects with no oral pathology^49^.

According to PCoA analysis, it was noted that supragingival plaques did not form well-separated clusters corresponding to smoking type or dental caries. Related community structures might be present in the supragingival plaques from different groups, which is consistent with the results of Xiao et al. who suggested that the bacterial structures in healthy and caries groups can be similar^20^. In spite of the presence of similar bacterial members in different samples, the relative abundances of these bacteria are possibly different as seen in this study.

One of the limitations of this study is the small sample size, due to the difficulty in recruiting subjects smoking a single tobacco type and fulfilling the inclusion criteria. However, our findings suggest that smoking negatively impacts the supragingival microbiome and support the need for additional research in a larger sample size. Large-scale follow-up studies are needed to prove the association between smoking and caries, and to show the contribution of the oral microbiome in the pathogenesis of caries in smokers. It is also essential to investigate the effects of other forms of smoking like smokeless tobacco in future studies. A shortcoming of caries detection method used in this study is the visual-tactile method which can overlook caries activity and non-cavitated caries. For both cost and practicality considerations, visual methods remain the standard for clinical assessment in dental practice. Other diagnostic methods to detect caries such as Fibre-optic Transillumination (FOTI), DIAGNOdent (DD), and Electrical Conductance (EC) can be utilized in non-cavitated caries detection to acquire a more comprehensive caries activity diagnosis in future studies^50^.

## Conclusion

To the best of our knowledge, our study is the first to shed light into the supragingival microbiome of smokers using different tobacco types and its relation to dental caries. We found that tobacco use had a significant impact on the composition of this microbiome. Cigarettes and alternative types of tobacco including medwakh and shisha significantly alter the supragingival plaque microbiome, and this effect is different in subjects with moderate-high caries compared to subjects with no or low dental caries.

Understanding the factors which alter the microbial community in the oral cavity and identifying keystone species in the oral microbiome is crucial to the design of health-promoting strategies to prevent dental caries. Further studies are required to test the impact of microbiota-based interventional measures on the oral health and possibility of their use for the prevention of dental caries by restoring a healthy oral microbiome.

## Supplementary Information

Supplementary Information 1.

Supplementary Information 2.

## Data Availability

Sequence data have been deposited in Sequence Read Archive, BioProject ID: PRJNA752710.
