# Supplementary material for: Supragingival microbiome alternations as a consequence of smoking different tobacco types and its relation to dental caries
Source: Sci Rep. 2022 Feb 21;12:2861. doi: 10.1038/s41598-022-06907-z (PMC8861055; doi:10.1038/s41598-022-06907-z)
Supplement: Supplementary file 1 — Supplementary Information 1. [file 41598_2022_6907_MOESM1_ESM.docx]

**Supplementary Table 1**. Demographic and clinical data for the participants. NC-LC= no or low caries ; MC-HC = moderate-high caries

| **ID** | **smoking** | **sex** | **age** | **ageBegin** | **duration of smoking years** | **VPI %** | **Decayed** | **Missing** | **Filled** | **DMF** | **Groups** |
| --- | --- | --- | --- | --- | --- | --- | --- | --- | --- | --- | --- |
| C4 | S-Cigarettes | Male | 23 | 15 | 8 | 0.1 | 1 | 1 | 4 | 6 | MC-HC |
| C12 | S-Cigarettes | Male | 35 | 26 | 9 | 0.29 | 2 | 3 | 0 | 5 | MC-HC |
| C25 | S-Cigarettes | Male | 37 | 15 | 22 | 0.41 | 6 | 1 | 0 | 7 | MC-HC |
| C30 | S-Cigarettes | female | 50 | 30 | 20 | 0.33 | 0 | 11 | 5 | 16 | MC-HC |
| C38 | S-Cigarettes | Male | 25 | 15 | 10 | 0.91 | 1 | 2 | 0 | 3 | NC-LC |
| C49 | S-Cigarettes | Male | 44 | 22 | 22 | 0.32 | 2 | 6 | 12 | 20 | MC-HC |
| C51 | S-Cigarettes | Male | 20 | 15 | 5 | 0.35 | 8 | 0 | 9 | 17 | MC-HC |
| C55 | S-Cigarettes | Male | 20 | 16 | 4 | 0.95 | 1 | 0 | 7 | 8 | MC-HC |
| C75 | S-Cigarettes | Male | 27 | 20 | 7 | 0.5 | 4 | 0 | 0 | 4 | NC-LC |
| C76 | S-Cigarettes | Male | 38 | 17 | 21 | 0.85 | 9 | 0 | 2 | 11 | MC-HC |
| M11 | S-Medwakh | Male | 24 | 17 | 7 | 0.11 | 4 | 0 | 0 | 4 | NC-LC |
| M17 | S-Medwakh | Male | 23 | 19 | 4 | 0.05 | 0 | 1 | 0 | 1 | NC-LC |
| M23 | S-Medwakh | Male | 24 | 22 | 2 | 0.47 | 0 | 0 | 8 | 8 | MC-HC |
| M29 | S-Medwakh | Male | 23 | 22 | 1 | 0.46 | 0 | 2 | 0 | 2 | NC-LC |
| M31 | S-Medwakh | Male | 28 | 26 | 2 | 0.74 | 3 | 0 | 0 | 3 | NC-LC |
| M52 | S-Medwakh | Male | 21 | 12 | 9 | 0.27 | 5 | 4 | 2 | 11 | MC-HC |
| M59 | S-Medwakh | Male | 18 | 10 | 8 | 0.22 | 0 | 1 | 4 | 5 | MC-HC |
| M64 | S-Medwakh | Male | 20 | 9 | 11 | 0.42 | 1 | 0 | 2 | 3 | NC-LC |
| M68 | S-Medwakh | Male | 27 | 14 | 13 | 0.8 | 6 | 2 | 2 | 10 | MC-HC |
| M77 | S-Medwakh | Male | 33 | 27 | 6 | 0.23 | 3 | 0 | 6 | 9 | MC-HC |
| SH14 | S-Shisha | Male | 24 | 19 | 5 | 0.22 | 1 | 0 | 11 | 12 | MC-HC |
| SH28 | S-Shisha | Male | 23 | 16 | 7 | 0.54 | 2 | 4 | 4 | 10 | MC-HC |
| SH42 | S-Shisha | Male | 54 | 52 | 2 | 0.57 | 2 | 1 | 2 | 5 | MC-HC |
| SH44 | S-Shisha | female | 27 | 22 | 5 | 0.4 | 0 | 4 | 1 | 5 | MC-HC |
| SH56 | S-Shisha | Male | 19 | 15 | 4 | 0.39 | 1 | 1 | 0 | 2 | NC-LC |
| SH60 | S-Shisha | Male | 20 | 18 | 2 | 0.14 | 8 | 0 | 0 | 8 | MC-HC |
| SH61 | S-Shisha | Male | 38 | 23 | 15 | 0.5 | 0 | 1 | 0 | 1 | NC-LC |
| SH62 | S-Shisha | Male | 44 | 24 | 20 | 0.19 | 0 | 3 | 1 | 4 | NC-LC |
| SH63 | S-Shisha | female | 20 | 18 | 2 | 0.13 | 0 | 0 | 11 | 11 | MC-HC |
| SH70 | S-Shisha | Male | 22 | 22 | 1 | 0.58 | 0 | 0 | 0 | 0 | NC-LC |
| X3 | Non-smoker | Male | 24 | 0 | 0 | 0.18 | 0 | 0 | 0 | 0 | NC-LC |
| X6 | Non-smoker | female | 47 | 0 | 0 | 0.11 | 4 | 4 | 7 | 15 | MC-HC |
| X15 | Non-smoker | Male | 62 | 0 | 0 | 0.3 | 0 | 0 | 2 | 2 | NC-LC |
| X16 | Non-smoker | female | 52 | 0 | 0 | 0.16 | 0 | 1 | 0 | 1 | NC-LC |
| X32 | Non-smoker | Male | 24 | 0 | 0 | 0.22 | 0 | 0 | 0 | 0 | NC-LC |
| X46 | Non-smoker | female | 34 | 0 | 0 | 0.76 | 0 | 0 | 8 | 8 | MC-HC |
| X84 | Non-smoker | Male | 23 | 0 | 0 | 0.75 | 0 | 1 | 0 | 1 | NC-LC |
| X87 | Non-smoker | Male | 29 | 0 | 0 | 0.37 | 3 | 6 | 0 | 9 | MC-HC |
| X89 | Non-smoker | Male | 44 | 0 | 0 | 0.14 | 0 | 1 | 2 | 3 | NC-LC |
| X92 | Non-smoker | Male | 46 | 0 | 0 | 0.02 | 3 | 0 | 1 | 4 | NC-LC |

**Supplementary Table 2**. The relative abundance (%) of phyla detected in the supragingival plaque samples from non-smokers, smokers of cigarettes, medwakh and shisha.

| Smoking group | Phyla |  |  |
| --- | --- | --- | --- |
|  |  | **Relative abundance (%)**  **Mean ± Std. Deviation** | |
| Non-smokers | *Firmicutes* | 51.22 ± | 22.66 |
|  | *Proteobacteria* | 26.24 ± | 22.11 |
|  | *Fusobacteria* | 8.01 ± | 6.11 |
|  | *Bacteroidetes* | 11.97 ± | 7.30 |
|  | *Candidatus Saccharibacteria* | 1.24 ± | 1.18 |
|  | *Actinobacteria* | 0.79 ± | 0.74 |
|  | *Spirochaetes* | 0.40 ± | 0.60 |
|  | *Tenericutes* | 0.06 ± | 0.07 |
| Cigarette’s smokers | *Firmicutes* | 65.56 ± | 16.17 |
|  | *Proteobacteria* | 18.02 ± | 10.31 |
|  | *Fusobacteria* | 7.14 ± | 4.72 |
|  | *Bacteroidetes* | 7.31 ± | 3.60 |
|  | *Candidatus Saccharibacteria* | 0.84 ± | 0.75 |
|  | *Actinobacteria* | 0.57 ± | 0.22 |
|  | *Spirochaetes* | 0.38 ± | 0.90 |
|  | *Tenericutes* | 0.10 ± | 0.14 |
| Medwakh smokers | *Firmicutes* | 65.30 ± | 14.90 |
|  | *Proteobacteria* | 14.77 ± | 9.88 |
|  | *Fusobacteria* | 8.95 ± | 2.70 |
|  | *Bacteroidetes* | 9.25 ± | 6.27 |
|  | *Candidatus Saccharibacteria* | 0.79 ± | 0.51 |
|  | *Actinobacteria* | 0.71 ± | 0.39 |
|  | *Spirochaetes* | 0.10 ± | 0.13 |
|  | *Tenericutes* | 0.04 ± | 0.03 |
| Shisha smokers | *Firmicutes* | 55.19 ± | 12.08 |
|  | *Proteobacteria* | 24.55 ± | 5.82 |
|  | *Fusobacteria* | 8.14 ± | 5.98 |
|  | *Bacteroidetes* | 10.01 ± | 4.90 |
|  | *Candidatus Saccharibacteria* | 0.78 ± | 0.82 |
|  | *Actinobacteria* | 0.94 ± | 0.45 |
|  | *Spirochaetes* | 0.19 ± | 0.22 |
|  | *Tenericutes* | 0.05 ± | 0.04 |

**Supplementary Table 3**. The relative abundance (%) of all species detected in the supragingival plaque samples from non-smokers, smokers of cigarettes, medwakh and shisha.

Included as an excel sheet


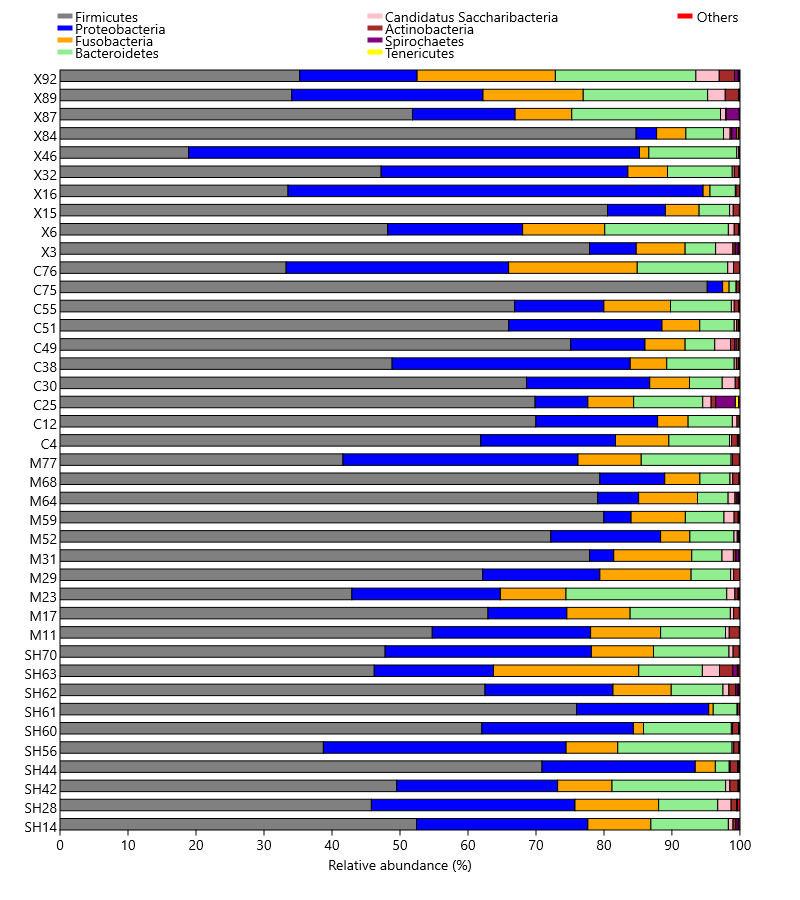


**Supplementary Figure 1. The relative abundance (%) of phyla detected in 40 supragingival plaque samples**

**A B**

**
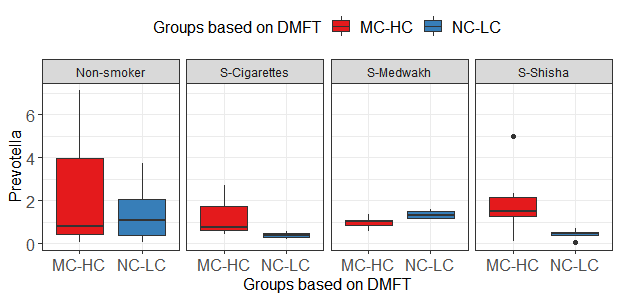

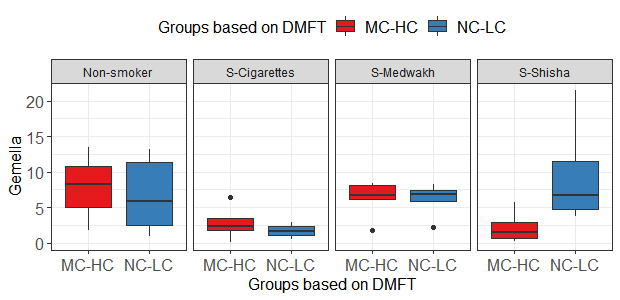
**

**C D**

**
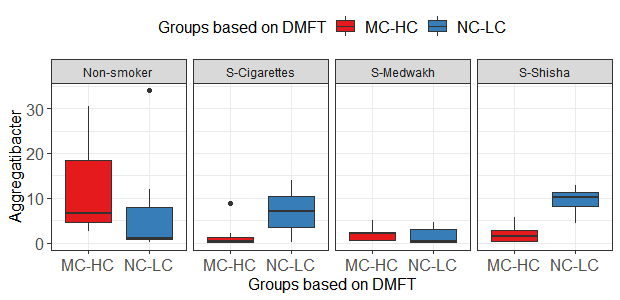

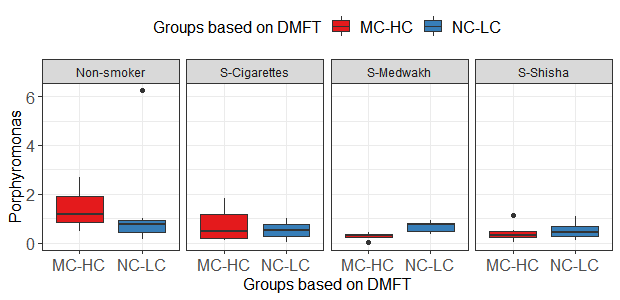
**

**E F**

**
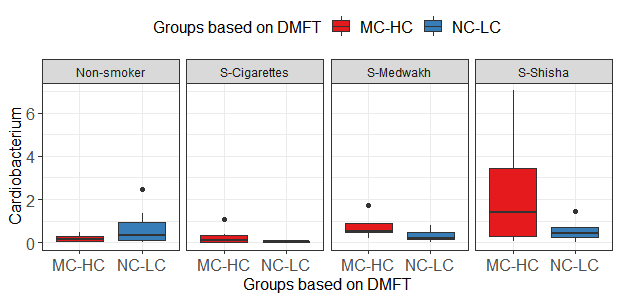

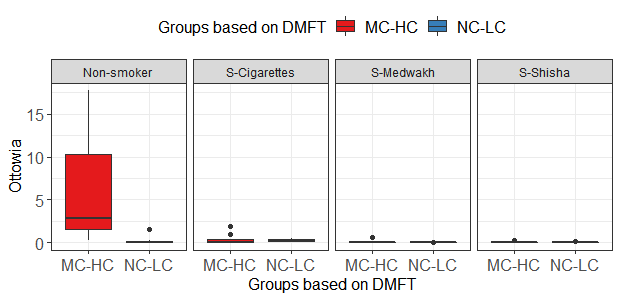
**

**G H**

**
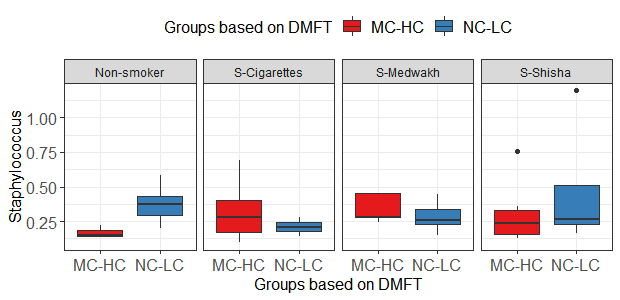

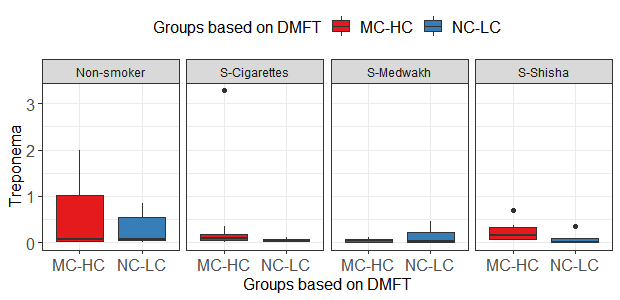
**

**I J**

**
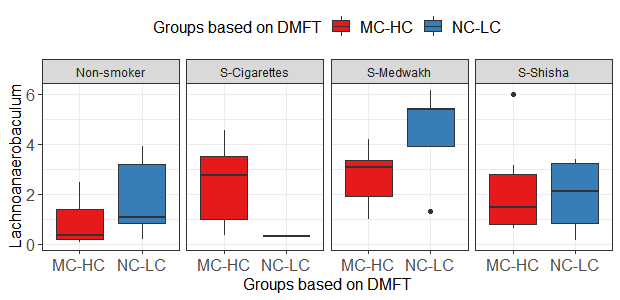

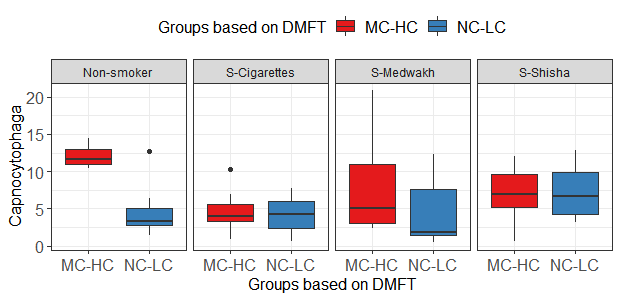
**

**K L**

**
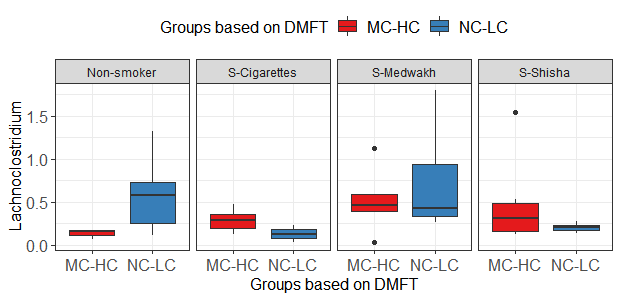

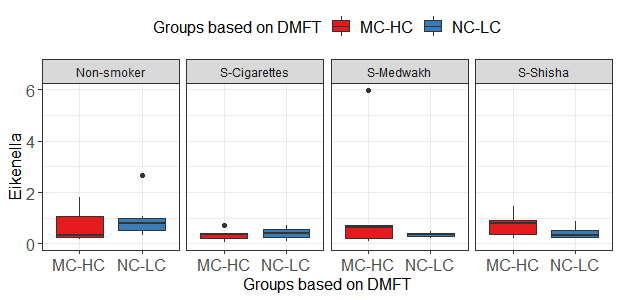
**

**M N**

**
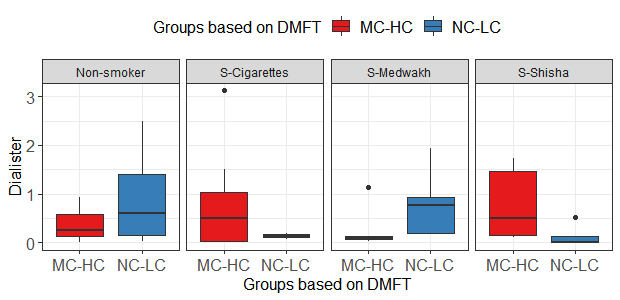

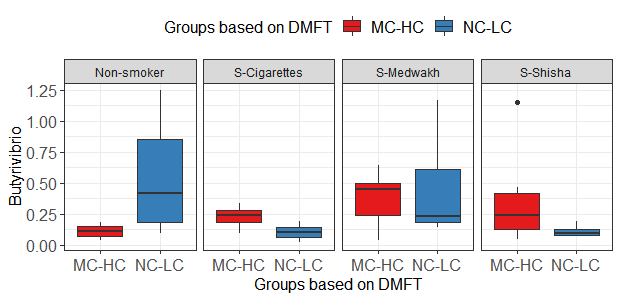
**

**O P**

**
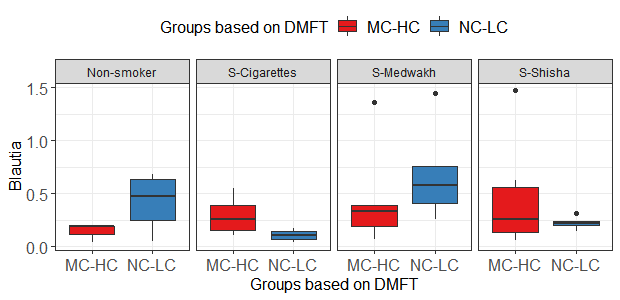

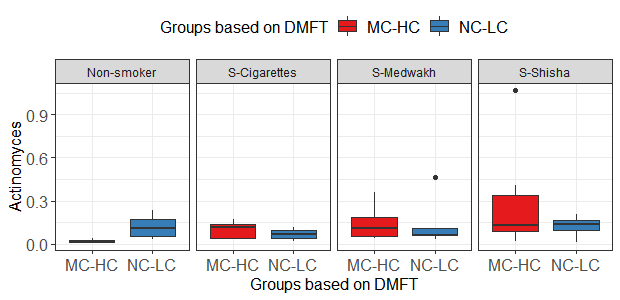
**

**Supplementary Figure 2.** Comparison of the relative abundance (%) of the major genera detected in the supragingival plaque samples; collected from subjects with no or low (NC-LC) caries and moderate-high (MC-HC) caries, in smokers of different types of tobacco and non-smokers. Box plots show Q1-median-Q3 with data range. Black dots are outlier values. Only the genera (n=16) that were significantly different between the study groups considering both smoking and caries are shown in the figure.

**A**

**
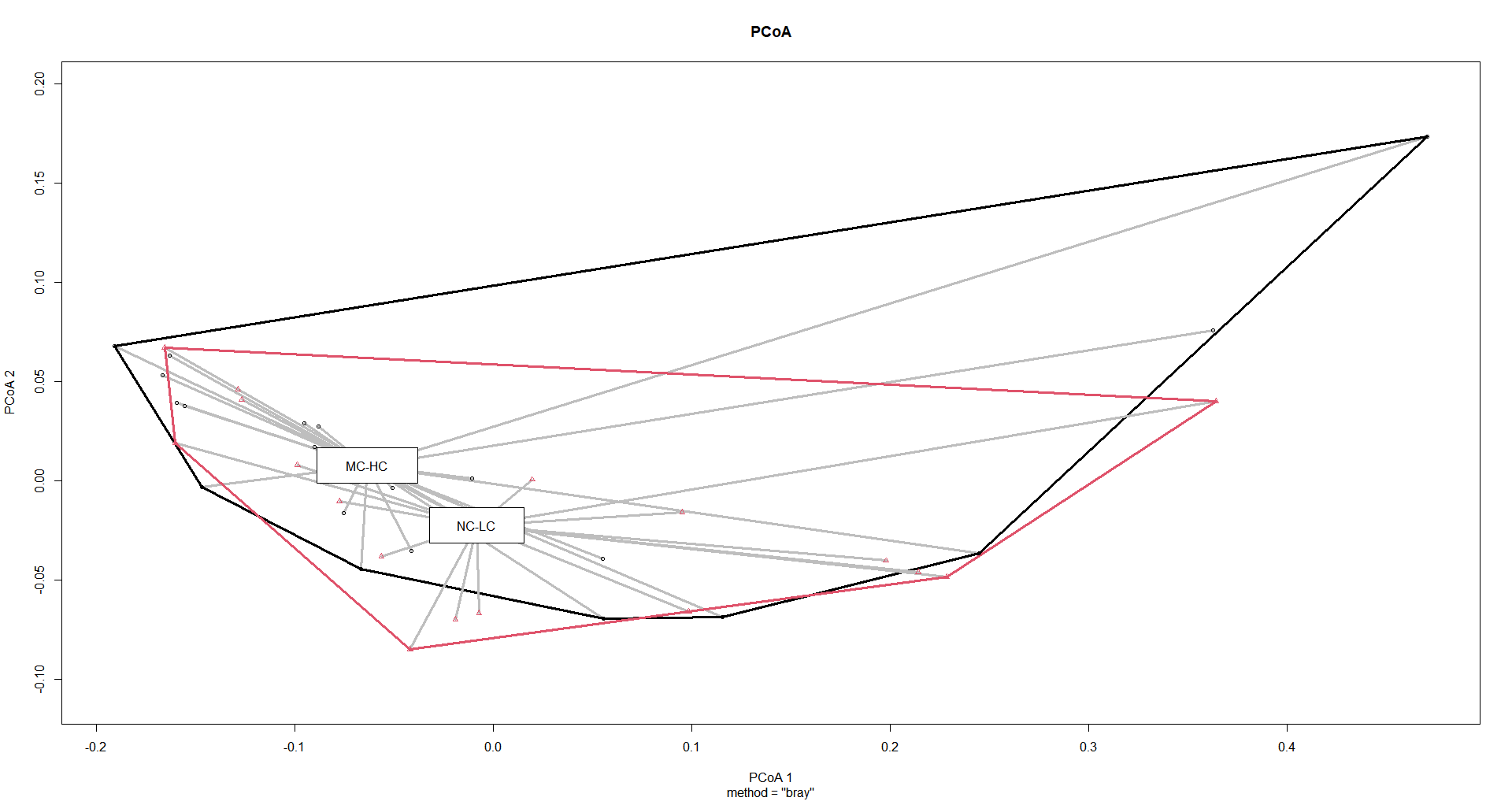
**

**B**


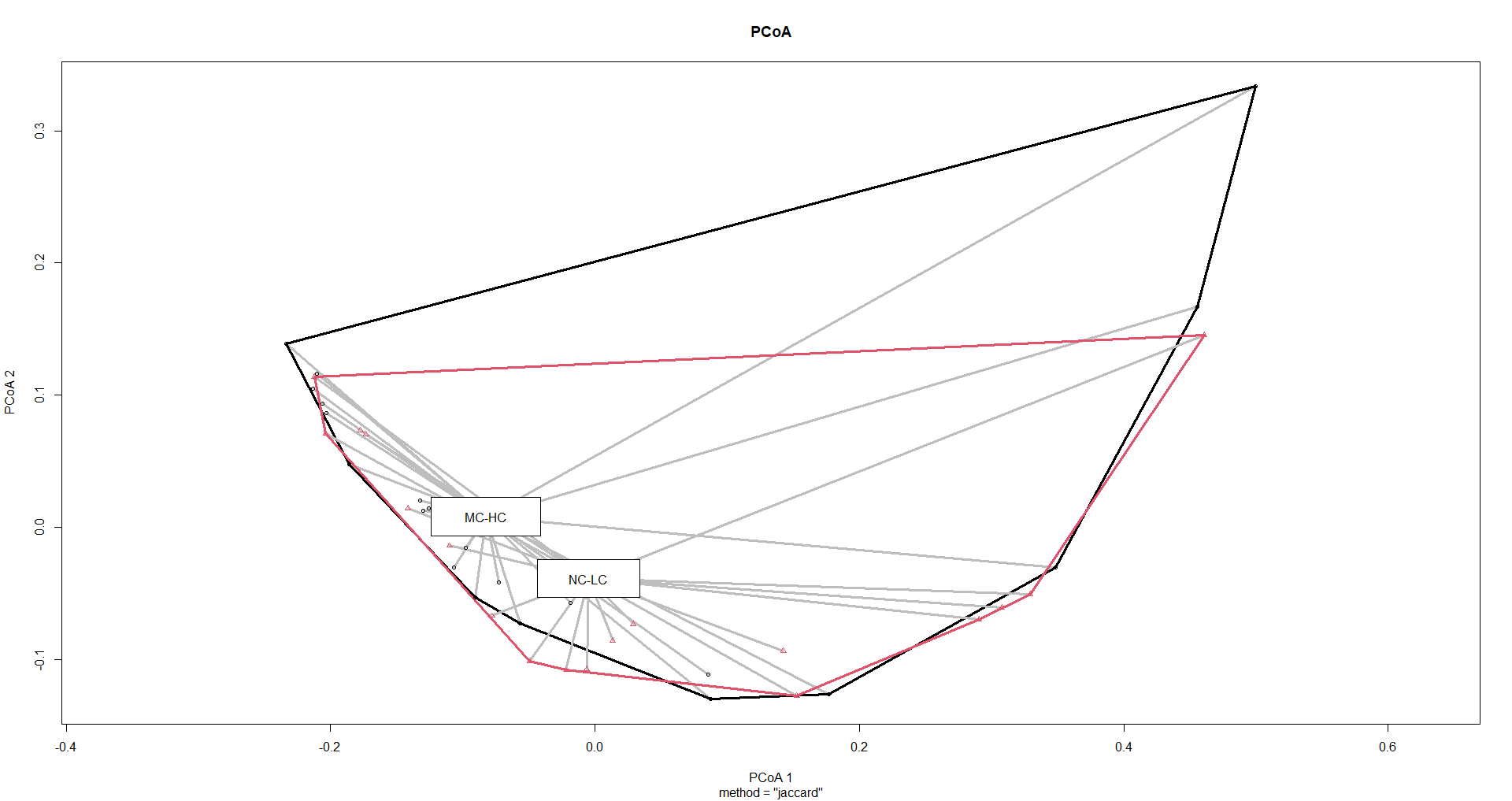


**C**

**
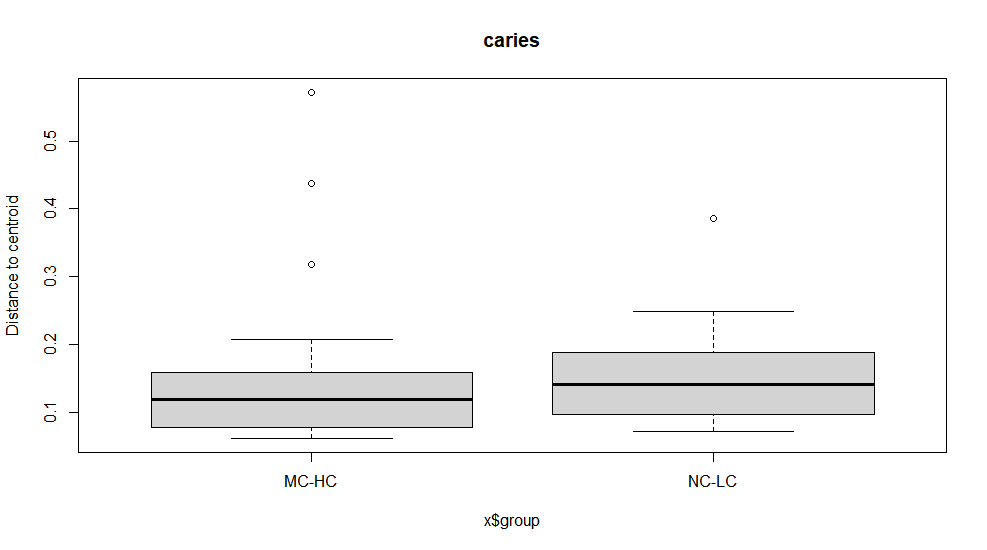
**

**D**

**
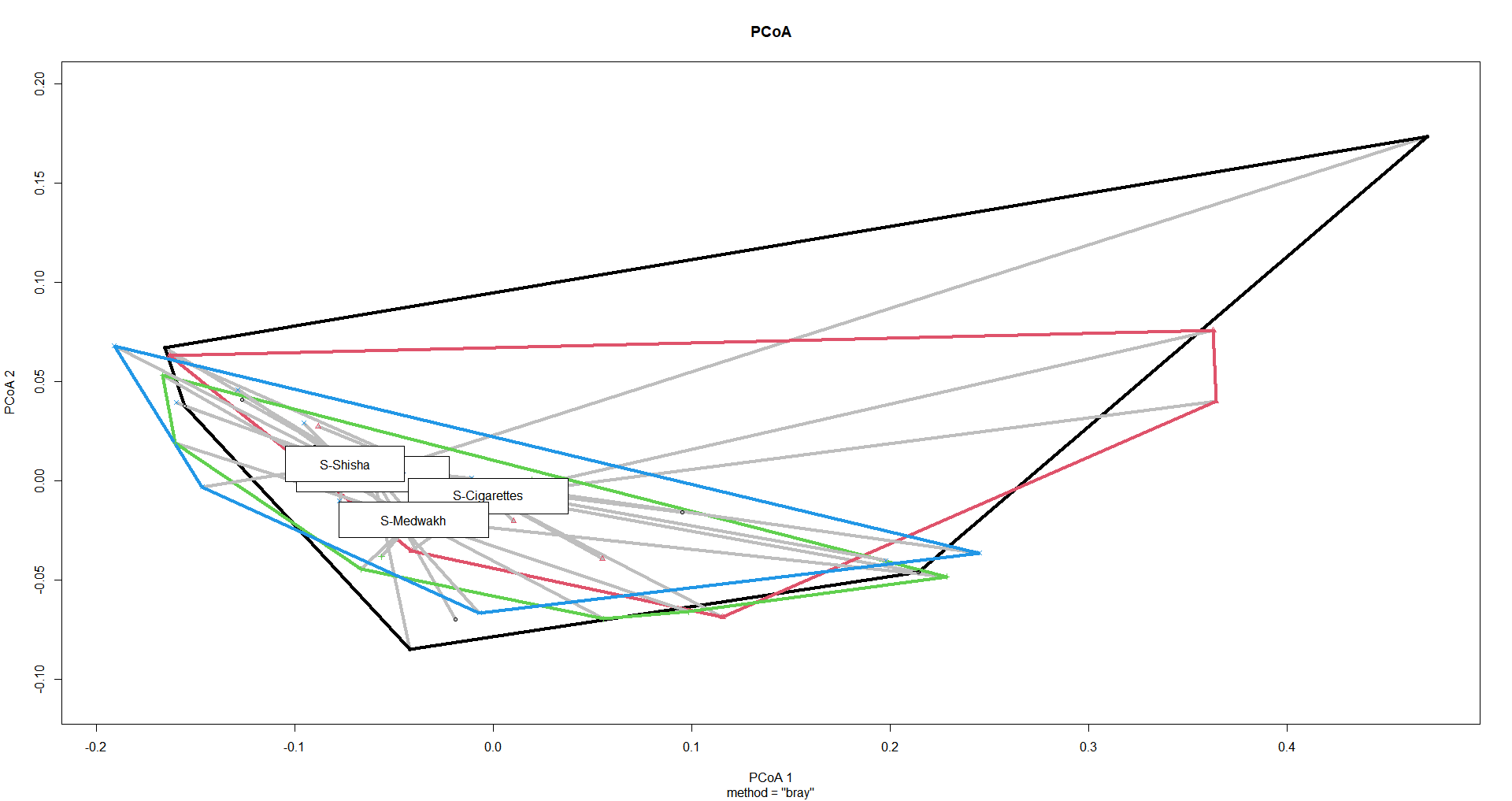
**

**E**


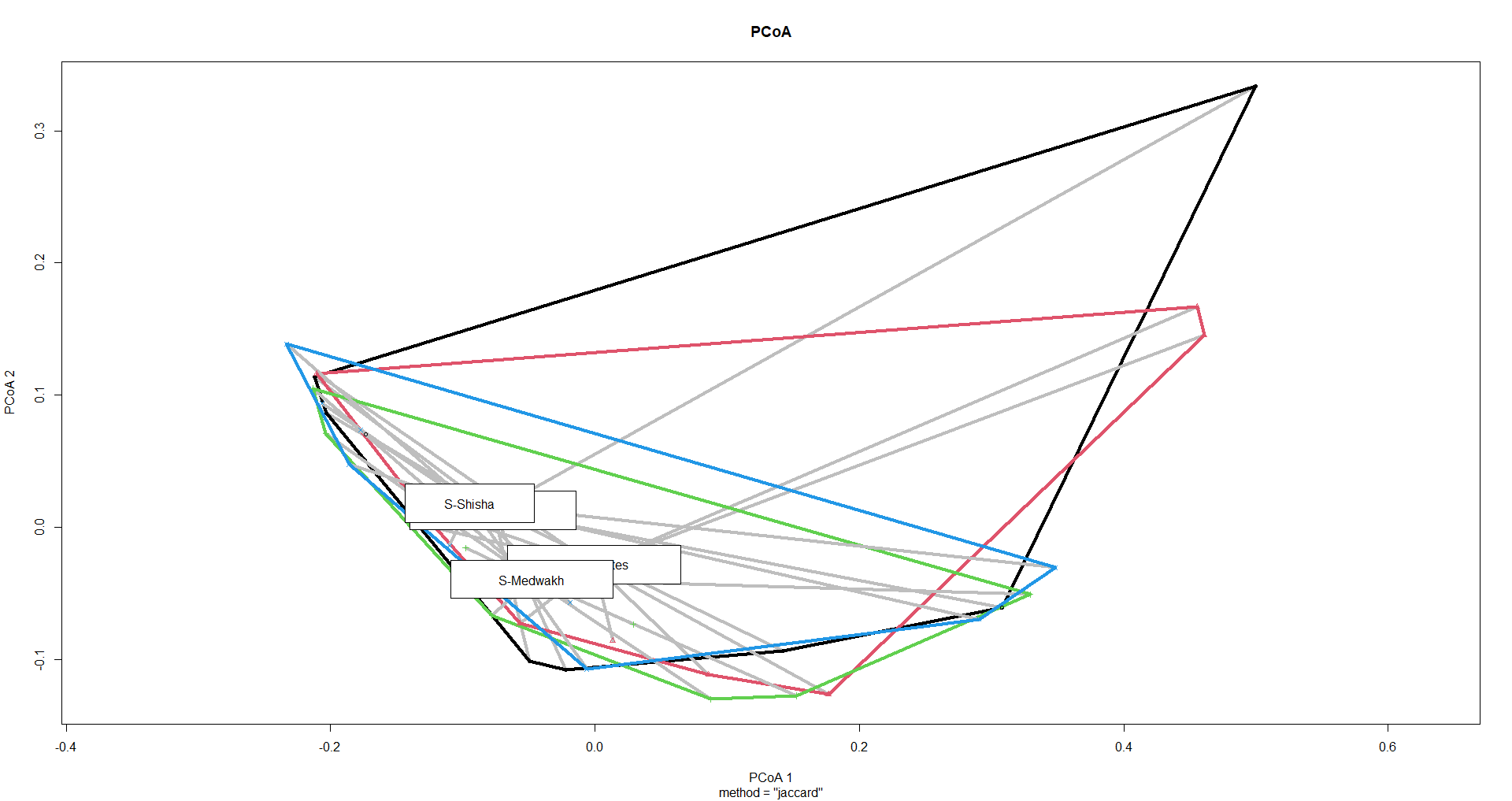


**F**


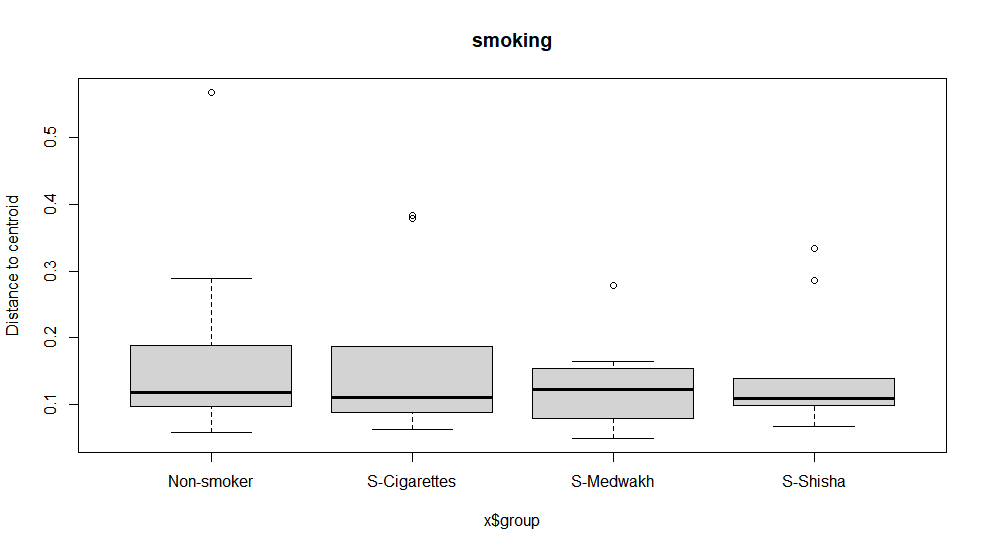


**Supplementary Figure 3. Beta diversity measured using Bray Curtis index (A and D) and Jaccard index (B and E). The samples were clustered based on the indices according to the dental caries (C) and tobacco smoking (F). No significant difference was found between the groups.**
